# Assessing running stability: a systematic review of methods

**DOI:** 10.1186/s13102-026-02062-4

**Published:** 2026-09-09

**Authors:** Cagla Kettner, Nicolai Fleischmann, Thorsten Stein

**Affiliations:** BioMotion Center, Institute of Sports and Sports Science, Karlsruhe Institute of Technology, Karlsruhe, Germany

**Keywords:** Locomotion, Movement variability, Nonlinear dynamics, Motor control, Lyapunov exponent, Uncontrolled manifold approach

## Abstract

**Background:**

Running stability is increasingly investigated in biomechanics and motor control research. However, the term is operationalized using diverse methods that may reflect different theoretical constructs, which complicates cross-study comparison and interpretation. This systematic methodological review aimed to identify, evaluate, and synthesize quantitative methods used to assess stability during running and to clarify the stability constructs represented by these methods.

**Methods:**

The review was conducted according to PRISMA 2020 and PERSiST guidelines. PubMed, Web of Science, Scopus, and Google Scholar were searched for English-language studies published or available online from 2000 to the final search date of 17 December 2025. Eligible studies were primary experimental studies involving human running tasks, biomechanical data, and a quantitative stability-related outcome. Non-running tasks, studies without original human running data, non-quantitative studies, modelling or simulation studies, reviews, conference papers, animal studies, and robotic studies were excluded. Methods were synthesized qualitatively and categorized according to their conceptual basis. Reporting quality was assessed using a structured quality assessment tool.

**Results:**

After screening and eligibility assessment, 43 studies were included. Stability was assessed using only linear methods in 9 studies, only nonlinear methods in 20 studies, combined linear and nonlinear methods in 7 studies, and coordination-based methods in 7 studies. Linear methods mainly quantified the magnitude of variability or discrete features of movement execution. Nonlinear methods primarily characterized the temporal structure of variability through measures of trajectory divergence, signal regularity, temporal correlations, and return behavior. Coordination-based methods examined the functional variability of elemental variables with respect to task-relevant performance variables. Reporting quality was moderate overall, with limited reporting of reliability, the number of analyzed strides, and method-specific processing details.

**Conclusions:**

The findings show that running stability is not represented by a single universal measure. Instead, different methods capture distinct aspects of movement variability, discrete features of movement execution, perturbation sensitivity, return behavior, or task-related coordination. Future studies should justify method selection according to the stability construct of interest and report key processing parameters to enhance reproducibility, interpretation, and comparability across studies.

**Trial registration:**

This systematic review was registered on the Open Science Framework prior to study screening (https://osf.io/e73xw).

**Supplementary Information:**

The online version contains supplementary material available at https://doi.org/10.1186/s13102-026-02062-4.

## Background

Running is one of the most widely practiced forms of physical activity worldwide and is associated with substantial health benefits [1–3]. Prospective cohort studies and meta-analytic evidence suggest that running participation is associated with lower risks of all-cause, cardiovascular, and cancer mortality, highlighting its public health relevance [1, 2, 4]. Despite these benefits, running-related musculoskeletal injuries are common, with a substantial proportion appearing to be overuse injuries affecting the lower extremity [5–7]. These injuries are consequential because they may restrict participation in sport and leisure activities, contribute to discontinuation of running, particularly among novice runners, and impose a measurable health and economic burden through healthcare utilization and work absenteeism [8–10].

Given the high prevalence and consequences of running-related injuries, growing attention has been directed toward biomechanical and motor control factors that may help explain how runners control movement in the presence of perturbations or changing task demands [11–13]. Within this context, stability has emerged as an important construct in running biomechanics and motor control research [11, 12, 14]. Stability-related constructs are frequently used to interpret how runners respond to small perturbations and how the control of running movement changes under different task constraints, including footwear, surface irregularity, fatigue, speed, and running experience [14–19]. In addition, recent work has linked whole-body dynamic stability to running economy [20]. Stability has also been examined from a coordination perspective, with studies investigating how movement variability is organized in a redundant system to stabilize task-relevant performance variables under factors such as running experience, fatigue, running speed, and shoe stack height [21–23]. However, despite its widespread use, stability does not refer to one consistently defined construct. As a result, the operationalization of stability varies across studies, and the term has been used to denote fundamentally different biomechanical and motor control constructs [24–27].

Against this background, it is important to clarify what is meant by stability from a biomechanical and motor control perspective. In the locomotion literature, stable gait has been pragmatically defined as gait that does not lead to falls despite perturbations [24]. More specific stability constructs, however, can be distinguished. Specifically, local dynamic stability refers to the sensitivity of the system to very small perturbations acting continuously over time, whereas orbital stability refers to whether a periodic gait pattern converges back toward its limit cycle from one step or stride to the next [28, 29]. Global stability, by contrast, refers to whether the system can withstand larger perturbations without falling, and is commonly characterized in terms of the basin of attraction of a stable gait pattern [28, 30]. Beyond these recovery-based notions, motor control approaches such as the uncontrolled manifold framework address stability in terms of how variability among redundant degrees of freedom is organized to stabilize a task-relevant performance variable [26, 31, 32]. Furthermore, in practice, greater movement variability is often interpreted as evidence of poorer stability, and variability-based measures have frequently been used to assess gait stability [33, 34]. This interpretation may be problematic because variability and stability are related but distinct properties of movement [25, 27, 35, 36]. Moreover, variability may also reflect flexibility of the system and exploration in response to changing task and environmental constraints [37–39]. These distinctions indicate that the assessment of running stability may require approaches that consider different properties of movement fluctuations, including their magnitude, temporal structure, and task-related function.

Accordingly, a range of methodological approaches has been used to assess stability. Linear measures such as the standard deviation and coefficient of variation quantify the magnitude of fluctuations in biomechanical signals, but they do not capture temporal organization, interdependence across strides, or how perturbations evolve over time [34, 40, 41]. Nonlinear methods derived from dynamical systems theory address these limitations by characterizing the temporal organization of movement [12, 35]. For example, Lyapunov exponents are used to quantify local dynamic stability by estimating how rapidly neighboring trajectories diverge, Floquet multipliers are used to quantify orbital stability by assessing return toward a periodic orbit from one step or stride to the next, and entropy-based measures quantify the regularity or predictability of the underlying time series [28, 29, 34, 42]. On the other hand, task-oriented motor control approaches address stability from a different perspective. For example, the uncontrolled manifold approach evaluates whether variability among elemental variables covaries to stabilize a task-relevant performance variable [22, 26, 32], whereas the Tolerance-Noise-Covariation method decomposes performance variability into tolerance, noise, and covariation components, which reflect the extent to which performance depends on the chosen solution, random variation around that solution, and compensatory coordination among variables, respectively [43–45]. In this framework, stability is primarily reflected by tolerance, describing the extent to which the chosen region of task space is resistant to motor noise or perturbations [43]. These methods are not interchangeable, because they quantify distinct properties of movement, including fluctuation magnitude, temporal organization, perturbation sensitivity, signal regularity, and task-relevant coordination.

Although the broader locomotion literature provides important conceptual and methodological foundations, much of the review literature on stability has focused on walking rather than running [24, 33]. Yet walking and running differ fundamentally in their biomechanics and motor control [46, 47]. Walking is commonly interpreted using an inverted-pendulum framework, and several stability constructs used in gait analysis, including the extrapolated center of mass and margin of stability, are derived from this mechanical model [48–50]. Running, in contrast, is more appropriately represented by a spring-mass model and exhibits self-stabilizing properties that arise from the interaction of leg stiffness, body dynamics, and ground contact mechanics [51–53]. Accordingly, stability measures developed for walking may not transfer directly to running, particularly when they rely on assumptions that are specific to inverted-pendulum walking dynamics. However, running-specific review literature remains relatively narrow; for example, Hunter et al. [12] focused on nonlinear methods used to assess changes in running movement dynamics, rather than the broader spectrum of linear, nonlinear, and coordination-based methods. To our knowledge, no systematic review has yet provided an integrated synthesis of the methods used to assess running stability, including the different stability constructs they address, the measures they use, and their methodological strengths and limitations.

Against this background, this systematic methodological review aims to identify, evaluate, and synthesize the methods used to assess stability during running. Specifically, it examines the reported stability measures, summarizes available information on reliability and validity where provided, and clarifies the stability constructs these methods represent. It also seeks to identify conceptual and methodological gaps in the current literature and to provide a clearer foundation for future research on running stability in relation to performance and injury.

## Methods

This study was conducted as a systematic methodological review [54] and reported in accordance with the “Preferred Reporting Items for Systematic Reviews and Meta-Analyses (PRISMA 2020)” [55] and “PRISMA in Exercise, Rehabilitation, Sport medicine and SporTs science (PERSiST)” Guidelines [56]. The review was registered on the Open Science Framework Registries (https://osf.io/e73xw) prior to study screening. No amendments were made to the registered review protocol.

The objective was to provide a comprehensive synthesis of the quantitative methods used to assess running stability. The study design followed the SDMO (Studies, Data, Methods, Outcomes) framework, which is particularly suited for method-oriented reviews [54]. The framework was applied as follows:

- Studies (S): Experimental studies conducted with human participants
- Data (D): Biomechanical data used to assess running stability
- Methods (M): Quantitative approaches used to operationalize stability
- Outcomes (O): Any reported stability-related measures, with no restrictions due to expected heterogeneity

### Data sources and search strategy

A systematic literature search was conducted in the following electronic databases: PubMed, Web of Science, Scopus, and Google Scholar. The search included English-language studies published or available online between 2000 and the final search date of 17 December 2025. Articles assigned to a 2026 volume or issue were eligible if they had already been published online and indexed by the final search date.

The search strategy was developed to combine terms related to stability, running, and biomechanical measurement, and was adapted to the syntax of each database. The core search string was as follows: *(*stability OR perturbation OR "postural control") AND (runn* OR athlet*) AND (biomechanic* OR kinematic* OR kinetic* OR "motion analysis")*.* Where supported by the database, additional exclusion terms were applied to reduce the retrieval of studies focused primarily on walking, gait, jumping, review articles, non-human models, or robotic systems: NOT (walk OR gait OR jump *OR* review OR animal* OR robotic*). In addition to the database search, the reference lists of relevant full-text articles were screened to identify additional eligible studies.

One reviewer (NF) performed the initial database search and removed duplicate records. Before screening, duplicate records and records marked as ineligible by automation tools were excluded. Two reviewers (CK and NF) independently screened the titles of all remaining records against the eligibility criteria. Records not excluded during title screening were subsequently screened by abstract. Full-text reports were then retrieved and independently assessed for eligibility by the same two reviewers. Additional records identified through citation searching were considered for inclusion according to the same eligibility criteria. Disagreements were resolved through discussion, with input from a third reviewer (TS) when necessary.

### Eligibility criteria

Studies were considered eligible for inclusion if they were primary experimental studies involving human participants. Eligible studies were required to investigate running as a distinct task, report biomechanical data, and include a quantitative method or metric used to operationalize running stability. To allow for a comprehensive methodological synthesis, no restrictions were applied regarding participant characteristics, running conditions, or specific stability-related outcome measures.

Studies were excluded if they focused exclusively on other locomotor or movement tasks, such as walking, jumping, standing balance, or postural control tasks not involving running. Studies involving sports with running components, such as football, basketball, or other field- and court-based sports, were excluded unless running was assessed as a distinct experimental task. Studies were also excluded if running stability was mentioned but not operationalized using a quantitative measure, or if they focused only on modelling, simulation, or the stability of a model rather than original human running data. Reviews, secondary articles, conference papers or abstracts, non-peer-reviewed publications, animal studies, and robotic studies were not considered.

### Data extraction and analysis

Data extraction was performed using a standardized approach. Extracted information included study and sample characteristics, experimental setup and running conditions, measurement procedures, biomechanical data collection and processing, modelling approaches, statistical analyses, and methods used to quantify running stability. Information on stability definition, reliability or validity tests, main findings, and practical or clinical interpretation were also included (Table 1). Information related to study aims, power analysis, sample characteristics, experimental setup and running conditions, reliability and validity assessment, statistical analyses, main findings, and practical or clinical interpretation was reported in the supplementary materials, as these items were not directly related to the assessment of running stability. Variables considered directly relevant to stability assessment are reported in the Results section and discussed subsequently.

**Table 1:** Data extraction items used in this systematic review

1	Study aim
2	Sample characteristics, including age, gender, height, body mass, and training status
3	Warm-up and familiarization procedures
4	Running task, including speed, duration, and surface condition
5	Measurement equipment
6	Biomechanical data collected
7	Data preprocessing, including filtering
8	Modeling procedures, including kinematic or kinetic calculations
9	Definition or conceptualization of stability
10	Method used to quantify running stability
11	Number of steps or strides analyzed
12	Statistical analyses, including dependent and independent variables
13	Reliability and validity information, if reported
14	Main findings related to running stability
15	Practical or clinical interpretation of the stability measure

Data extraction was performed independently by two reviewers (CK and NF). Extracted data were compared between reviewers, and discrepancies were resolved through discussion. Adjudication by the third reviewer (TS) was not required.

Because this review focused on methodological synthesis, all quantitative biomechanical approaches used to assess running stability were extracted, regardless of the specific variable or measurement technique used. Methods were synthesized qualitatively and grouped according to their main operational principles and underlying stability construct.

If sufficient homogeneity in methods and reported outcomes was identified, a meta-analysis was considered. However, due to the heterogeneity in study designs, biomechanical variables, and stability metrics, the primary analytical approach was descriptive and comparative.

### Quality assessment

The reporting quality of the included studies was assessed using a structured reporting quality assessment tool adapted from Winter et al. [57]. The tool was modified to reflect the methodological focus of the present review and to evaluate reporting completeness, methodological transparency, and reproducibility in studies assessing running stability (Table 2). It was not designed as a formal risk of bias assessment. Each criterion was scored as 0, 1, or 2, indicating that the criterion was not fulfilled, partly fulfilled or insufficiently reported, or clearly fulfilled, respectively. The quality assessment was conducted to evaluate the methodological transparency and reproducibility of the included studies and to identify common strengths and limitations in the assessment of running stability.

**Table 2:** Reporting quality assessment criteria used in this systematic review

Item	Criterion
Q1	Are there clearly defined research questions?
Q2	Was a power analysis conducted to determine the required sample size?
Q3	Were participant demographics clearly defined?
Q4	Were inclusion and exclusion criteria clearly defined?
Q5	Were adequate familiarization or practice trials provided prior to data collection?
Q6	From the information provided on the experimental protocol used, could the research be replicated?
Q7	Were the methods used to quantify running stability clearly explained?
Q8	Were the data used for quantifying running stability clearly defined?
Q9	Was test–retest reliability performed or appropriately referenced?

Quality assessment was performed independently by two reviewers (CK and NF). The reviewers were not blinded to study authorship; however, the same procedures were applied to all studies, including those authored by members of the review team. NF was not an author of any included study authored by members of the review team and therefore provided an independent second assessment. The level of agreement between the reviewers before discussion was not formally quantified or recorded. Discrepancies were resolved through discussion between CK and NF, and adjudication by the third reviewer (TS) was not required.

## Results

### Search results and study selection

**Fig. 1:**
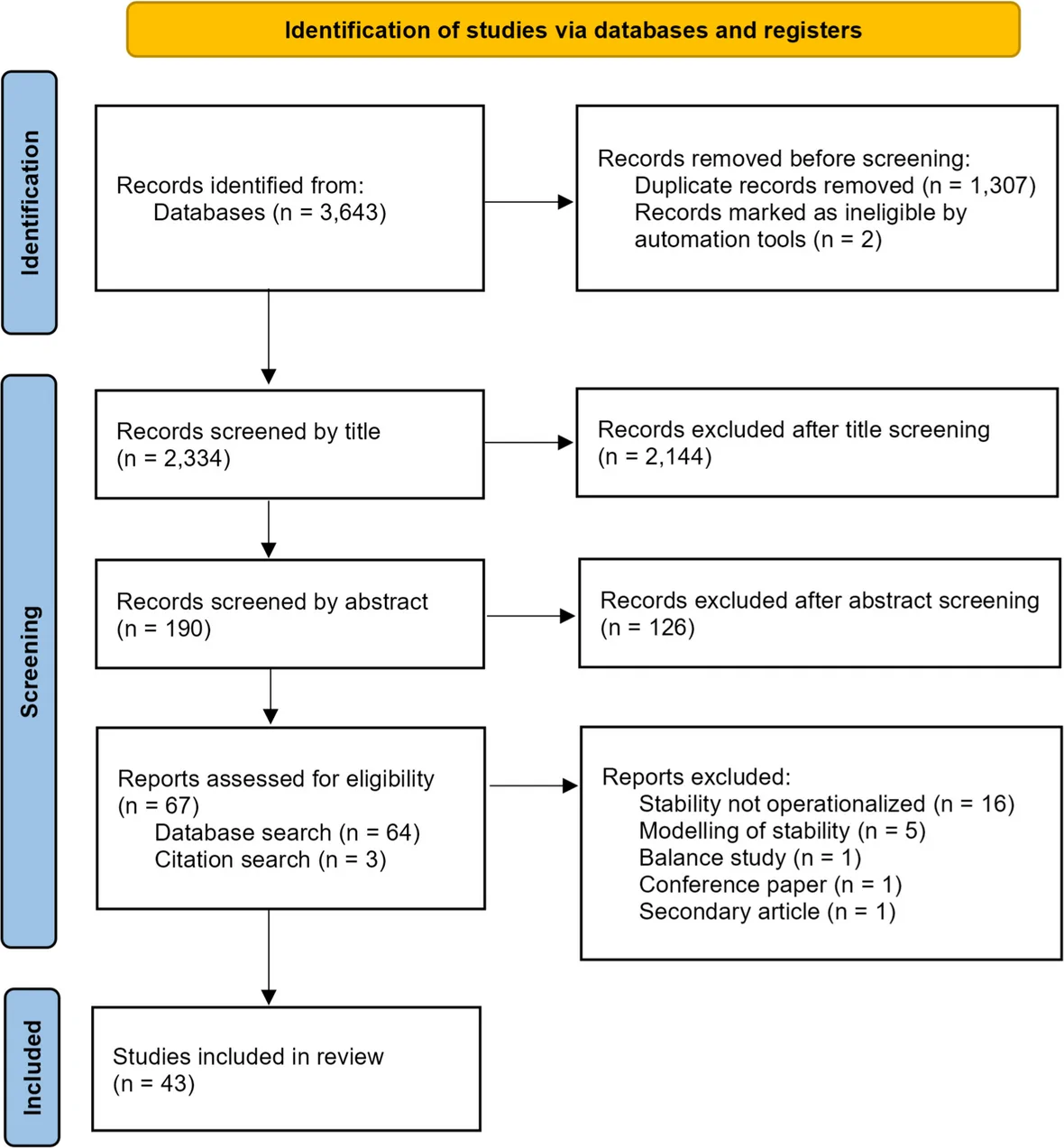
Flow diagram of the systematic search and study selection process. Adapted from the PRISMA 2020 flow diagram template for systematic reviews [55]

The database searches identified 3,643 records: 1,289 from PubMed, 997 from Web of Science, 1,279 from Scopus, and 78 from Google Scholar. Google Scholar results were ranked by relevance, and screening proceeded through the first 78 results; subsequent results were consistently unrelated to the review question and eligibility criteria. After removing duplicates and records marked as ineligible by automation tools, 2,334 records were screened by title. Of these, 190 records proceeded to abstract screening, and 67 reports were assessed for full-text eligibility, including 3 reports identified through citation searching. After full-text assessment, 43 studies were included in the review (Fig. 1).

**Fig. 2:**
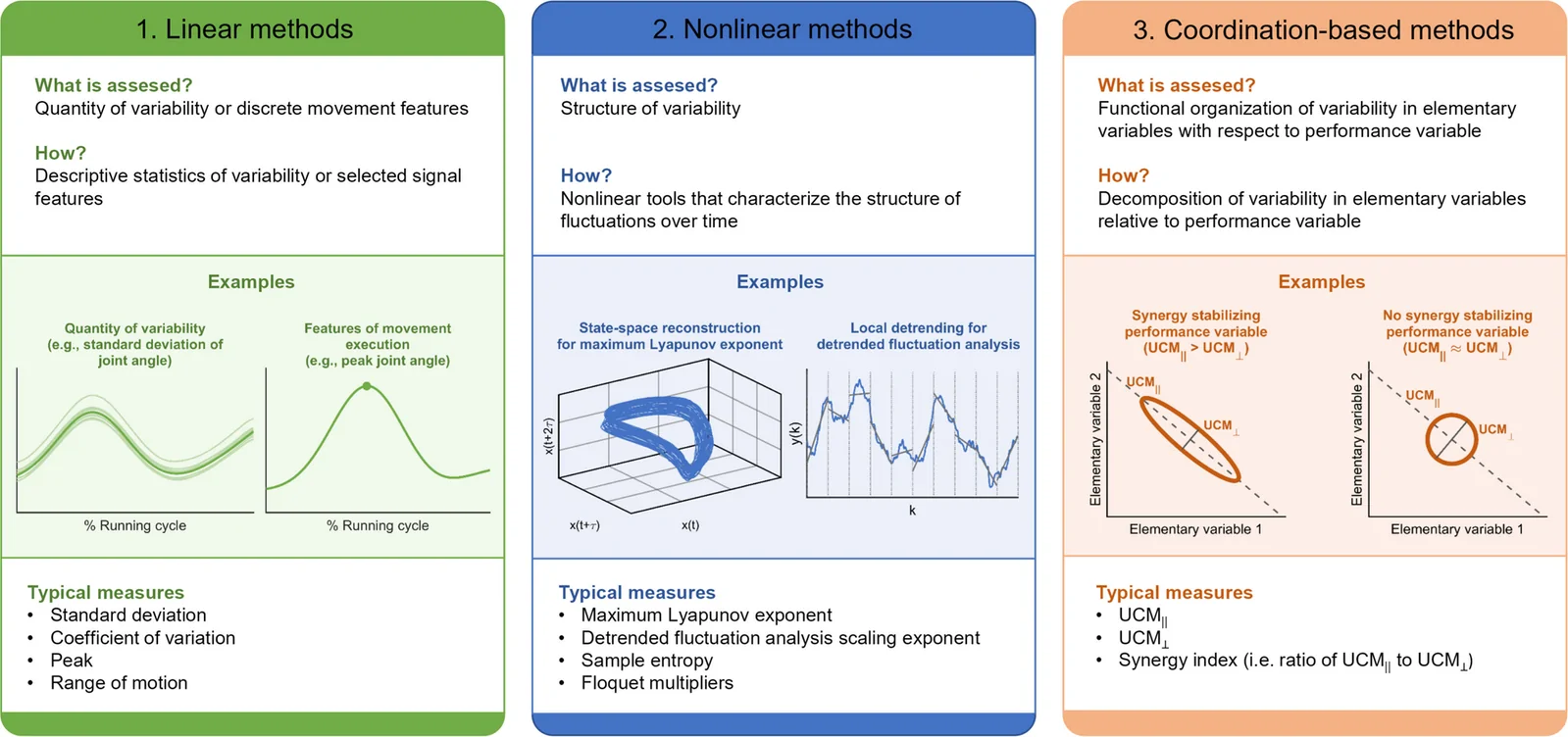
The identified methods were grouped into linear, nonlinear, and coordination-based methods according to their conceptual basis. The examples shown are illustrative and do not represent all possible methods within each category. Abbreviations: UCM, uncontrolled manifold; UCM∥, variability parallel to the uncontrolled manifold; UCM⊥, variability orthogonal to the uncontrolled manifold

Following study selection, the methods used to quantify running stability were categorized into three main groups according to their conceptual basis: linear methods, nonlinear methods, and coordination-based methods. As some studies applied more than one methodological approach, the study counts for the linear and nonlinear groups were not mutually exclusive. Of the 43 included studies, 9 used only linear methods, 20 used only nonlinear methods, 7 used both linear and nonlinear methods, and 7 used coordination-based methods. For the method-specific synthesis, the 7 studies combining linear and nonlinear approaches were included in both relevant sections. Consequently, 16 studies were considered in the linear methods section and 27 in the nonlinear methods section, while the 7 coordination-based studies were discussed separately. An overview of the conceptual basis, representative examples, and typical measures of these three methodological groups is provided in Fig. 2.

Additional study information not directly related to the assessment of running stability is provided in the supplementary materials. Power analysis and sample characteristics are reported in Supplementary Table 1. Experimental setup, running conditions, reliability and validity assessment, and statistical analyses are reported in Supplementary Table 2. Study aims, main findings, and practical or clinical interpretations are summarized in Supplementary Table 3.

### Linear methods

Among the 16 studies classified as using linear methods, stability was most commonly defined as dynamic stability (*n* = 9, Table 3). Other definitions included rearfoot stability [58, 59], ankle stability [60], body stability [61], locomotor stability [62], and stability of coordination patterns [63]. Running protocols varied in duration or distance, ranging from short overground running trials of 10–90 m to treadmill or running protocols lasting from 1 min to approximately 14 min, or until volitional exhaustion (Table 4). Trial duration or distance was not specified in two studies [64, 65].

**Table 3:** Linear methods used to assess running stability in the included studies. The table summarizes the stability definition, method used to operationalize stability, variable to which the method was applied, and additional methodological details reported

Study	Stability definition	Method used	Applied to	Details
Aristizábal Pla et al., 2021 [66]	Dynamic stability	RMS ratio and autocorrelation coefficient	3D acceleration of trunk	NS
Ataabadi et al., 2021 [61]	Body stability	Standard deviation	3D angles of hip, knee and ankle	Mean across stride
Hagen et al., 2025 [58]	Rearfoot stability	Value at IC, ROM, peak velocity	Frontal foot angle	NS
Hilário et al., 2022 [67]	Dynamic stability	Peak value	Excursions of internal rotation and adduction of hip, and external rotation and abduction of knee	NS
Iosa et al., 2013 [62]	Locomotor stability	RMS, harmonic ratio, ratio index	3D acceleration of trunk	NS
Jordan et al., 2009 [63]	Coordination pattern stability	Standard deviation	Relative phases of sagittal ankle-hip and ankle-knee angle pairs	NS
Kelly et al., 2022 [64]	Dynamic stability	Peak value	Center of pressure time to contact	NS
Kettner et al., 2025a [60]	Ankle stability	Total time at a value, peak values	Frontal foot angle	During stance
Law et al., 2018 [68]	Dynamic stability	Total displacement; ROM, peak value and peak velocity	ML and AP GRF, COP; frontal foot angle	NS
Rethwilm et al., 2021 [69]	Dynamic stability	Margin of stability	Difference of the extrapolated COM trajectory and the base of support	NS
Schütte et al., 2016 [70]	Dynamic stability	RMS	RMS ratio: 3D acceleration of trunk; Autocorrelation coefficients: step and stride	NS
Schütte, Sackey, et al., 2018 [71]	Dynamic stability	RMS ratio and autocorrelation coefficient	RMS ratio: 3D acceleration of trunk; Autocorrelation coefficients: step and stride	NS
Schütte, Seerden, et al., 2018 [19]	Dynamic stability	RMS ratio and autocorrelation coefficient	RMS ratio: 3D acceleration of trunk; Autocorrelation coefficients: step and stride	NS
Sterzing et al., 2015 [59]	Rearfoot stability	Value at IC, ROM, peak velocity	Frontal foot angle	During stance
Wang & Yuan, 2024 [65]	Ankle stability	Peak value, peak speed and peak torque	Frontal foot angle and torque	During stance
Zhang et al., 2024 [72]	Dynamic stability	Total displacement	ML and AP COP	During stance

*Abbreviations AP* Anterior–posterior, *COM* Center of mass, *COP* Center of pressure, *GRF* Ground reaction force, *IC* Initial contact, *ML* Mediolateral, *NS* Not specified, *RMS* Root mean square, *ROM* Range of motion

**Table 4:** Methodological characteristics of studies using linear methods to assess running stability**.** The table summarizes the running duration or distance per condition, measurement devices, data collected, sampling frequency, filtering procedures, data-processing information, normalization procedures, and number of analyzed cycles. Sampling frequency and filtering procedures are reported for the variables used to operationalize stability

Study	Duration or distance per condition	Measurement devices	Data collected	Frequency (Hz)	Filter	Data processing	Normalization	No cycles
Aristizábal Pla et al., 2021 [66]	12 min	IMUs on trunk	3D acceleration of trunk	1,000	BW, low-pass, 4th, 50 Hz (signals)	NS	NS	NS
Ataabadi et al., 2021 [61]	2 min	IMUs on trunk and lower body	3D kinematics of segments and joints	200	BW, low-pass, 4th, 8 Hz (signals)	Kinematics	Stride	NS
Hagen et al., 2025 [58]	5 × 15 m	Electrogoniometer; force plates	Rearfoot eversion	1,000	NS	NS	NS	NS
Hilário et al., 2022 [67]	3 × 1 min	Infrared cameras, 16 lower body and hip markers	Marker movements	120	NS	Kinematics	NS	NS
Iosa et al., 2013 [62]	10 m	IMU on trunk	3D acceleration of trunk	100	Low pass, 20 Hz (signals)	NS	NS	4 strides
Jordan et al., 2009 [63]	5 min	Infrared cameras; 22 markers on head, trunk and lower body	Marker movements	125	Zero-phase, 2nd, 6 Hz (markers)	Kinematics	NS	NS
Kelly et al., 2022 [64]	NS	Infrared cameras; markers on head, trunk and lower body; force plates	Marker movements and COP	200 (markers), 1,000 (force)	BW, low-pass, 4th, 10 Hz (markers), 50 Hz (force)	Kinematics	NS	NS
Kettner et al., 2025a [60]	90 s	Infrared cameras; 65 markers on full-body	Marker movements	200	BW, low-pass, 4th, 10 Hz (markers)	Kinematics	NS	20 strides
Law et al., 2018 [68]	5 × 15 m	Infrared cameras; markers on foot; force plates	Marker movements and GRF	240 (markers), 1,600 (force)	BW, low-pass, 4th, 12 Hz (markers), 100 Hz (GRF)	NS	NS	NS
Rethwilm et al., 2021 [69]	13 m	Infrared cameras; markers on trunk and lower body; force plates	Marker movements and GRF	NS	NS	NS	NS	NS
Schütte et al., 2016 [70]	90 m	IMU on trunk	3D acceleration of trunk	1024	BW, low-pass, 4th, 50 Hz (signals)	NS	NS	10 strides
Schütte, Sackey, et al., 2018 [71]	until exhaustion	IMU on trunk	3D acceleration of trunk	1024	BW, low-pass, 4th, 50 Hz (signals) and unfiltered	NS	NS	20 strides
Schütte, Seerden, et al., 2018 [19]	13.63 ± 1.86 min and 14.14 ± 1.79 min	IMUs on trunk	3D acceleration of trunk	1024	BW, low-pass, 4th, 50 Hz (signals)	NS	NS	10 strides
Sterzing et al., 2015 [59]	5 × 16 m	Infrared cameras; markers on hip and lower body; force plates	Marker movements and GRF	200	BW, low-pass, 4th, 30 Hz (kinematics)	Kinematics	NS	NS
Wang & Yuan, 2024 [65]	NS	Infrared cameras; force plates	Marker movements and GRF	200	BW, low-pass, 13 Hz (markers)	Kinematics, kinetics	NS	NS
Zhang et al., 2024 [72]	5 × 15 m	Infrared cameras; markers on full-body; force plates	COP	1,000	BW, low-pass, 4th, 50 Hz (force)	Kinetics	NS	NS

*Abbreviations BW* Butterworth, *COM* Center of mass, *COP* Center of pressure, *GRF* Ground reaction force, *Hz* Hertz, *IMU* Inertial measurement unit, *NS* Not Specified

Kinematic data were collected using optical motion-capture systems, inertial measurement units (IMUs), or electrogoniometry (Table 4). Six studies used IMUs, mainly positioned on the trunk [19, 61, 62, 66, 70, 71], whereas nine studies used optical motion-capture systems with marker sets placed on the foot, lower body, hip, trunk, or full body [59, 60, 63–65, 67–69, 72]. One study used an electrogoniometer to assess rearfoot motion [58]. Force or pressure data were recorded using force plates in seven studies [58, 59, 64, 65, 68, 69, 72]. The collected data included trunk acceleration, marker trajectories, rearfoot eversion, ground reaction forces, center of pressure data, and joint or segment kinematics (Table 4).

Sampling frequencies ranged from 100 to 1,024 Hz for kinematic data. Studies including kinetic data reported separate sampling frequencies of 1,000 or 1,600 Hz. Filtering procedures were reported in most studies and generally consisted of low-pass Butterworth filtering, with cut-off frequencies ranging from 6 to 50 Hz for kinematic data and from 50 to 100 Hz for kinetic data (Table 4). Three studies did not specify filtering procedures [58, 67, 69]. Kinematic data processing was reported in seven studies [59–61, 63, 65, 67, 72], whereas kinetic processing was reported only in two studies [65, 72]. Normalization procedures were generally not reported; one study normalized data to the stride cycle [61]. The number of analyzed cycles was specified in five studies, ranging from 4 to 20 strides [19, 60, 62, 70, 71], whereas the remaining studies did not report the number of cycles used (Table 4).

Linear stability was quantified using several approaches, including peak-value measures [58–60, 65, 67, 68], range of motion [58, 59, 68], displacement-based measures [68, 72], root mean square–based measures [19, 62, 66, 70, 71], autocorrelation coefficients [19, 66, 70, 71], standard deviation [61], harmonic ratio and mean ratio index [62], relative phase variability [63], minimum time to contact [64], and margin of stability [69]. These methods were applied to trunk acceleration [19, 62, 66, 70, 71], lower body angles [58–61, 63, 65, 67, 68], step and stride patterns [19, 70, 71], center of pressure [64, 68, 72], ground reaction forces [68], and the relationship between extrapolated center of mass trajectory and base of support [69].

### Nonlinear methods

Among the 27 studies classified as using nonlinear methods, the most frequently used stability definition was local dynamic stability (*n* = 14; Table 5). Other definitions included dynamic stability [19, 66, 70, 71, 73, 74], running stability [13, 75–77], global system stability [78], global stability [60], body stability [61], gait stability [79], and local orbital stability [80]. Running protocols varied in duration or distance, ranging from short 30 s recordings to several minutes per condition, prolonged running trials, 5,000 m or 10 km runs, and running until exhaustion (Table 6). One study did not specify trial duration or distance [75]. Kinematic data were collected using optical motion-capture systems (*n* = 13), or IMUs (*n* = 12; Table 6), whereas two studies did not collect kinematic data [75, 78]. Kinetic data were recorded using instrumented treadmills in four studies [14, 78, 79, 81], pressure-sensing insoles in two studies [76, 82], and an unspecified force-measurement system in one study [75]. One study also included electromyographic recordings [83]. The collected data included marker trajectories, trunk acceleration, segment angular velocity, joint kinematics, underfoot pressure, ground reaction forces, and muscle activity (Table 6).

**Table 5:** Nonlinear methods used to assess running stability in the included studies**.** The table summarizes the stability definition, method used to operationalize stability, variable to which the method was applied, and methodological details

Study	Stability definition	Method used	Applied to	Details
Agresta et al., 2019 [78]	Global system stability	DFA	Step frequency and contact time	NS
Aristizábal Pla et al., 2021 [66]	Dynamic stability	SampEn	Steps and stride	NS
Arshi et al., 2015 [73]	Dynamic stability	MLE and SampEn	MLE: movement of trunk; SampEn: distance foot to pelvis at IC and TO	MLE: AMI, FNN, RS, short-term, continuous; SampEn: m = 2; r = 0.2
Ataabadi et al., 2021 [61]	Body stability	MLE	3D angles of hip, knee and ankle	AMI, FNN, RS, short-term, continuous
Blum et al., 2011 [75]	Running stability	Poincaré map	Vertical movement of COM	NS
Cerrito et al., 2025 [84]	Local dynamic stability	MLE	Sagittal angle of hip, knee and ankle	AMI, FNN, Wolf, continuous
Ekizos et al., 2017 [14]	Local dynamic stability	MLE	Vertical movement of trunk	AMI, FNN, RS, short-term, continuous
Ekizos et al., 2018 [81]	Local dynamic stability	MLE	Vertical movement of trunk	AMI, FNN, RS, continuous
Fohrmann et al., 2023 [13]	Running stability	MLE	3D angular velocity of trunk, hip, tibia and foot	AMI, FNN, RS, short-term, continuous (treadmill) and non-continuous (overground)
Fohrmann et al., 2025 [76]	Running stability	MLE	3D angular velocity of tibia and foot	AMI, FNN, RS, short-term, continuous
Frank et al., 2019 [17]	Local dynamic stability	MLE	Sagittal angle of hip, knee and ankle	AMI, FNN, Wolf, continuous
Hoenig et al., 2019 [85]	Local dynamic stability	MLE	3D angular velocity of trunk, hip and foot	AMI, FNN, RS, short-term, continuous
Hollander et al., 2021 [86]	Local dynamic stability	MLE	3D angular velocity of tibia	AMI, FNN, RS, short-term, continuous
Jordan et al., 2009 [63]	Local dynamic stability	MLE	Vertical movement of head and ankle	AMI, FNN, RS, long-term
Kettner et al., 2025a [60]	Local dynamic stability (MLE), global stability (DFA)	MLE and DFA	MLE: vertical movement of head, trunk, hip and foot; DFA: stride time	MLE: AMI, FNN, RS, short-term, continuous; DFA: bin size 4–24
Look et al., 2013 [74]	Dynamic stability	MLE	3D movement of hip; sagittal angle of hip and knee	AMI, FNN, Kantz (similar to RS), short-term, continuous
Mehdizadeh et al., 2014 [87]	Local dynamic stability	MLE	3D movement of trunk	AMI, FNN, RS, short-term and long-term, continuous
Mehdizadeh et al., 2016 [80]	Local dynamic stability (MLE), local orbital stability (maxFM)	MLE, maxFM	AP and ML movement of ankle	MLE: AMI, FNN, RS, short-term, continuous; maxFM: peak over all Poincaré sections
Ogaya et al., 2024 [79]	Gait stability	MLE	Sagittal and frontal angle of ankle, sagittal angle of knee, 3D angles of hip and trunk	AMI, FNN, Rosenstein, continuous
Promsri, 2022 [77]	Running stability	MLE	Principal positions	AMI, FNN, Wolf, continuous
Santuz et al., 2018 [18]	Local dynamic stability	MLE	Vertical movement of trunk	AMI, FNN, RS, short-term, continuous
Schütte et al., 2016 [70]	Dynamic stability	SampEn	3D acceleration of trunk	NS
Schütte, Sackey, et al., 2018 [71]	Dynamic stability	SampEn	3D acceleration of trunk	NS
Schütte, Seerden, et al., 2018 [19]	Dynamic stability	SampEn	3D acceleration of trunk	NS
von Diecken et al., 2025 [20]	Local dynamic stability	MLE	3D angular velocity of forearm, shoulder, pelvis and tibia together	AMI, FNN, RS, short-term, continuous
Walsh, 2021 [83]	Local dynamic stability	MLE	3D acceleration of trunk; muscle synergies	AMI, FNN, RS, short-term, continuous
Xiang et al., 2022 [82]	Local dynamic stability	MLE	3D angular velocity of tibia and foot	AMI, FNN, RS, short-term, continuous

*Abbreviations 3D* Three-dimensional, *AMI* Average mutual information, *AP* Anterior–posterior, *COM* Center of mass, *DFA* Detrended fluctuation analysis, *FNN* Global false nearest neighbours, *IC* Initial contact, *maxFM* maximum Floquet multiplier, *ML* Mediolateral, *MLE* Maximum Lyapunov exponent, *NS* Not specified, *RS* Rosenstein’s algorithm, *SampEn* Sample entropy, *TO* Toe-off

**Table 6:** Methodological characteristics of studies using nonlinear methods to assess running stability**.** The table summarizes the running duration or distance per condition, measurement devices, data collected, sampling frequency, filtering procedures, data-processing information, normalization procedures, and number of analyzed cycles. Sampling frequency and filtering procedures are reported for the variables used to operationalize stability

Study	Duration or distance per condition	Measurement devices	Data collected	Frequency (Hz)	Filter	Data processing	Normalization	No cycles
Agresta et al., 2019 [78]	5 min	Instrumented treadmill	Underfoot pressure	120	NS	NS	NS	NS
Aristizábal Pla et al., 2021 [66]	12 min	IMUs on trunk	Triaxial acceleration of trunk	1,000	BW, low-pass, 4th, 50 Hz (signals)	NS	NS	NS
Arshi et al., 2015 [73]	2 min	Infrared cameras; trunk and foot markers	Marker movements	100	BW, low-pass, 4th, 10 Hz (markers)	NS	MLE: 10,000 data points; SampEn: unit variance	100 strides
Ataabadi et al., 2021 [61]	2 min	IMUs on trunk, hip, thigh, tibia and foot	3D kinematics of segments and joints	200	BW, low-pass, 4th, 8 Hz (kinematics)	Kinematics	5,000 data points	50 strides
Blum et al., 2011 [75]	NS	Force measurements but equipment NS	GRF	NS	NS	NS	NS	Total 2867 strides
Cerrito et al., 2025 [84]	10 min	Infrared cameras, hip and lower body markers	Marker movements	200	BW, low-pass, 2nd, 15 Hz (joint angles)	Kinematics	NS	100 strides
Ekizos et al., 2017 [14]	2 min	High-speed video cameras; 5 markers on back; instrumented treadmill	Marker movements and GRF	190	BW, low-pass, 4th, 20 Hz (markers)	NS	20,000 data points	278 steps
Ekizos et al., 2018 [81]	2 min	High-speed video cameras; 11 markers on back; instrumented treadmill	Marker movements and GRF	191	BW, low-pass, 4th, 20 Hz (markers)	NS	19,809 data points	279 steps
Fohrmann et al., 2023 [13]	Treadmill: 5 min; overground: 500 m	IMUs on trunk, hip, tibia and foot	3D angular velocity of trunk, hip, tibia and foot	128	No filter	NS	10,000 data points	100 strides
Fohrmann et al., 2025 [76]	3 min	IMUs on tibia and foot; pressure-sensing insoles	3D acceleration and angular velocity of tibia and foot	500	No filter	NS	10,000 data points	100 strides
Frank et al., 2019 [17]	4 min	Infrared cameras, marker clusters on hip, thigh, tibia and foot	Marker movements	100	BW, dual-pass, 2nd, 15 Hz (markers)	Kinematics	NS	216 strides
Hoenig et al., 2019 [85]	5,000 m	IMUs on trunk, hip and foot	3D angular velocity of trunk, hip and foot	100	NS	NS	15,000 data points	150 strides
Hollander et al., 2021 [86]	15 min	IMU on tibia	3D angular velocity of tibia	256	No filter	NS	10,000 data points	100 strides
Jordan et al., 2009 [63]	5 min	Infrared cameras; 22 markers on head, trunk and lower body	Marker movements	125	BW, 2nd, 6 Hz (markers)	Kinematics	NS	NS
Kettner et al., 2025a [60]	90 s	16 infrared cameras; 65 markers on full-body	Marker movements	200	BW, low-pass, 4th, 10 Hz (marker)	Kinematics	MLE: 10,000 points	100 strides
Look et al., 2013 [74]	8–10 strides	Infrared cameras; markers on hip and lower body	Marker movements	300	NS	Kinematics	NS	8 to 10 strides
Mehdizadeh et al., 2014 [87]	2 min	Infrared cameras; trunk marker	Marker movements	100	No filter	NS	10,000 data points	100 strides
Mehdizadeh et al., 2016 [80]	2 min	Infrared cameras; foot markers	Marker movements	100	No filter	NS	MLE: 10,000 data points; maxFM: 0–100% stride	MLE: 100 strides; maxFM: NS
Ogaya et al., 2024 [79]	30 s	Infrared cameras; 39 full-body markers; instrumented treadmill	Marker movements and GRF	100	BW, low-pass, 10 Hz (angles)	Kinematics	NS	30 strides
Promsri, 2022 [77]	30 s	Infrared cameras; 39 full-body markers	Marker movements	250	BW, low-pass, 3rd, 5 Hz (principal movements)	NS	NS	NS
Santuz et al., 2018 [18]	2 × 2 min	Infrared cameras; 10 markers on trunk and lower body	Marker movements	300	BW, low-pass, 4th, 20 Hz (markers)	NS	~ 33,000 data points	287 steps
Schütte et al., 2016 [70]	90 m	IMU on trunk	3D acceleration of trunk	1024	No filter	NS	NS	10 strides
Schütte, Sackey, et al., 2018 [71]	Until exhaustion	IMU on trunk	3D acceleration of trunk	1024	BW, low-pass, 4th, 50 Hz (signals) and no filter	NS	NS	20 strides
Schütte, Seerden, et al., 2018 [19]	13.63 ± 1.86 min; and 14.14 ± 1.79 min	IMU on trunk	3D acceleration of trunk	1024	No filter	NS	NS	10 strides
von Diecken et al., 2025 [20]	5 × 3 min	IMUs on full-body	3D angular velocity of forearms, shoulder, pelvis and tibia	100	NS	NS	10,000 data points	100 strides
Walsh, 2021 [83]	3 min	IMU on trunk; EMG electrodes on trunk and lower body	3D acceleration of trunk; activity of lower body and back muscles	IMU: 100; EMG: 1,000	IMU: BW, low-pass, 2nd, 20 and 2 Hz (signals); EMG: BW, band-pass, 2nd, 50–450 Hz, and low-pass, 2nd, 15 Hz (signals)	NS	IMU: 12,000 data points; EMG: 6,000 data points	IMU: 120 strides; EMG: 30 strides
Xiang et al., 2022 [82]	10 km at 11.2 ± 1.2 km/h	IMUs on tibia and foot; pressure-sensing insoles	3D angular velocity of tibia and foot	100	BW, low-pass, 2nd, 40 Hz (signals)	NS	8,000 data points	80 strides

*Abbreviations 3D* Three-dimensional, *BW* Butterworth, *EMG* Electromyography, *GRF* Ground reaction force, *Hz* Hertz, *IMU* Inertial measurement unit, *maxFM* maximum Floquet multiplier, *MLE* Maximum Lyapunov exponent, *NS* Not specified, *SampEn* Sample entropy

Sampling frequencies ranged from 100 to 1,024 Hz for kinematic data (Table 6). Seven studies reported that no filtering was applied [13, 19, 70, 76, 80, 86, 87], five did not specify filtering [20, 74, 75, 78, 85], and the remaining studies reported low-pass Butterworth or related filtering procedures with cut-off frequencies between 5 and 50 Hz (Table 6). Kinematic data processing was reported in seven studies [17, 60, 61, 63, 74, 79, 84], whereas kinetic processing was not specified in any of the studies.

Normalization procedures were reported in 16 studies, most commonly in studies using maximum Lyapunov exponent analyses (Table 6). In these studies, time series were typically normalized to a fixed length, often calculated as the number of analyzed strides × 100 data points. One study normalized the input data for sample entropy analysis to unit variance [73], and one study normalized maximum Floquet multiplier analysis to 0–100% of the stride cycle [80]. The number of analyzed cycles was specified in most studies. The most common approach was to analyze 100 strides [13, 20, 60, 73, 76, 80, 84, 86, 87], although other studies used 8–10 strides [74], 10 strides [19, 70], 20 strides [71], 30 strides [79, 83], 50 strides [61], 80 strides [82], 120 strides [83], 150 strides [85], 216 strides [17], 278–287 steps [14, 18, 81], or 2,867 strides [75]. Four studies did not specify the number of cycles analyzed [63, 66, 77, 78].

Nonlinear stability was most frequently quantified using the maximum Lyapunov exponent (n = 21; Table 5). Other methods included sample entropy [19, 66, 70, 71, 73], detrended fluctuation analysis [60, 78], Poincaré maps [75], and maximum Floquet multipliers [80]. Some studies combined methods, such as maximum Lyapunov exponent with sample entropy [73], detrended fluctuation analysis [60], or maximum Floquet multipliers [80].

The maximum Lyapunov exponent was applied to variables such as trunk movement [14, 18, 60, 73, 81, 87], head movement [60, 63], hip movement [60, 74], ankle movement [63, 80] or foot movement [60]; lower-body angles [17, 61, 74, 79, 84], segment angular velocities [13, 20, 76, 82, 85, 86], trunk acceleration [83], and muscle synergies [83].

For movement-based variables, the vertical component was most commonly analyzed [14, 18, 60, 63, 75, 81]. Sample entropy was applied to trunk acceleration [19, 70, 71], step and stride variables [66], and foot-pelvis center of mass distance measures [73]. Detrended fluctuation analysis was applied to step frequency [78], contact time [78], or stride time [60]. Poincaré-map analysis was applied to vertical center of mass movement [75], and maximum Floquet multipliers were applied to anterior–posterior and mediolateral ankle movement [80].

Methodological details were most commonly reported for maximum Lyapunov exponent calculations. Studies frequently used continuous time-series data, applied average mutual information to determine the time delay, used global false nearest neighbours to determine the embedding dimension, and applied Rosenstein’s algorithm for short-term maximum Lyapunov exponent analysis (Table 5). Some studies used Wolf’s algorithm [17, 77, 84], Kantz’s algorithm [74], or long-term Lyapunov exponent calculations [63, 87]. Where reported, sample entropy parameters included embedding dimension and tolerance [73]. For detrended fluctuation analysis, one study reported bin sizes [60].

### Coordination-based methods

Seven studies were classified as using coordination-based methods. Six studies defined stability as performance-variable stability [21–23, 88–90], whereas one study used the term system stability [91] (Table 7). Trial durations ranged from 15 s to 5 min per condition, with one fatigue protocol lasting 6.18 ± 2.45 min (Table 8). All studies used optical motion-capture systems, with markers placed on the lower body and hip [88, 91], full body [21–23, 90], or with marker placement not further specified [89]. Three studies additionally collected ground reaction force data using instrumented treadmills, although these data were not used as primary stability-related variables but for gait event detection [88, 89, 91].

**Table 7:** Methodological characteristics of studies using coordination-based methods to assess running stability. The table summarizes the running duration or distance per condition, measurement devices, data collected, sampling frequency, filtering procedures, data-processing information, normalization procedures, and number of analyzed cycles. Sampling frequency and filtering procedures are reported for the variables used to operationalize stability

Study	Duration or distance per condition	Measurement devices	Data collected	Frequency (Hz)	Filter	Data processing	Normalization	No cycles
Dias et al., 2025 [88]	3 min 30 s	Infrared cameras; markers on hip and lower body; instrumented treadmill	Marker movements and GRF	150	BW, low-pass, 4th, 10 Hz (markers)	Kinematics	Swing phase	38–43
Garofolini et al., 2024 [91]	5 min	Infrared cameras; 21 markers on hip and lower body; instrumented treadmill	Marker movements and GRF	250	BW, low-pass, 4th, 15 Hz (markers)	NS	40 ms before and after IC	40
Kettner et al., 2025b [21]	90 s	Infrared cameras; 65 markers on full-body	Marker movements	200	BW, low-pass, 4th, 10 Hz (markers)	Kinematics	Stance phase	20
Liew et al., 2024 [89]	30 s	Infrared cameras, markers; instrumented treadmill	Marker movements and GRF	150	BW, low-pass, 4th, 12 Hz (markers)	Kinematics	Stance phase	29–56
Möhler et al., 2019 [22]	15 s	Infrared cameras; 41 markers on full-body	Marker movements	200	BW, low-pass, 4th, 10 Hz (markers)	Kinematics	Stride	20
Möhler et al., 2020 [23]	1 min	Infrared cameras; 41 markers on full-body	Marker movements	200	BW, low-pass, 4th, 10 Hz (markers)	Kinematics	Stride	20
Möhler et al., 2022 [90]	6.18 ± 2.45 min	Infrared cameras; 42 markers on full-body	Marker movements	200	BW, low-pass, 2nd, 15 Hz (markers)	Kinematics	Stride	35

*Abbreviations BW* Butterworth, *GRF* Ground reaction force, *Hz* Hertz, *IC* Initial contact, *NS* Not specified

Coordination-based methods provided a distinct functional perspective on running stability. In contrast to linear methods, which mainly quantify the amount of variability, and nonlinear methods, which describe its temporal structure [27], coordination-based methods address the problem of motor redundancy by examining whether variability among elemental variables is organized in a way that stabilizes a task-relevant performance variable [26, 38, 114–116]. Thus, these methods address the functional role of variability rather than treating variability only as error or noise [117]. In the present review, all coordination-based studies used the uncontrolled manifold approach (Table 8). This method decomposes variability into components that do not affect the selected performance variable and components that do affect it [26]. A larger proportion of variability within the uncontrolled manifold than orthogonal to it is interpreted as evidence that the performance variable is stabilized by covariation among elemental variables [26].

**Table 8:** Coordination-based methods used to assess running stability in the included studies. The table summarizes the stability definition, method used, elemental and performance variables, and model details

Study	Stability definition	Method used	Elemental and performance variables	Details
Dias et al., 2025 [88]	Performance variable stability	UCM	EV: sagittal lower body joint angles and frontal pelvis angle (7 DoF), PV: vertical foot position (1 DoF)	Multiple regression analyses
Garofolini et al., 2024 [91]	System stability	UCM	EV: sagittal lower body joint angles (4 DoF), PV: horizontal and vertical leg position (2 DoF)	Geometric model
Kettner et al., 2025b [21]	Performance variable stability	UCM	EV: 3D full-body joint angles (44 DoF), PV: center of mass position (3 DoF)	Anthropometric model
Liew et al., 2024 [89]	Performance variable stability	UCM	EV: leg length and angle (2 DoF), PV: center of mass position (2 DoF)	Geometric model
Möhler et al., 2019 [22]	Performance variable stability	UCM	2D model, EV: sagittal lower body angles (4 DoF), PV: center of mass position (2 DoF); 3D model: EV, 3D full-body joint angles (50 DoF), PV: center of mass position (3 DoF)	2D geometric model and 3D anthropometric model
Möhler et al., 2020 [23]	Performance variable stability	UCM	EV: 3D full-body joint angles (50 DoF), PV: center of mass position (3 DoF)	3D anthropometric model
Möhler et al., 2022 [90]	Performance variable stability	UCM	EV: 3D full-body joint angles (50 DoF), PV: center of mass position (3 DoF)	3D anthropometric model

*Abbreviations 2D* Two-dimensional, *3D* Three-dimensional, *DoF* Degrees of freedom, *EV* Elemental variable, *PV* Performance variable, *UCM* Uncontrolled manifold

Sampling frequencies ranged from 150 to 250 Hz (Table 7). Filtering procedures were reported in all studies and consisted of low-pass Butterworth filtering of marker data, most commonly fourth-order filtering at 10 Hz [21–23, 88]. Other reported filter settings included fourth-order filtering at 12 [89] or 15 Hz [91] and second-order filtering at 15 Hz [90]. Kinematic data processing was reported in six studies [21–23, 88–90]; one study did not specify the kinematic processing software [91].

All coordination-based studies used the uncontrolled manifold approach. Normalization was performed relative to specific gait phases or analysis windows, including stride cycle [22, 23, 90], stance phase [21, 89], swing phase [88], or a 40 ms window before and after initial contact [91]. The number of analyzed cycles was reported in all studies and ranged from 20 to 56 (Table 7).

The uncontrolled manifold method was applied using elemental variables such as lower-body joint angles [22, 88, 91], full-body joint angles [21–23, 90], or leg length and leg angle [89]. Performance variables included center of mass position in two [22, 89] or three dimensions [21–23, 90], vertical foot position [88], or horizontal and vertical leg position [91]. Anthropometric models [21–23, 90], geometric models [22, 89, 91], or multiple regression analyses [88] were used to define the relationship between elemental and performance variables.

### Quality assessment

The reporting quality assessment is presented in Supplementary Table 4. The mean quality score across the 43 included studies was 57%, with individual study scores ranging from 33 to 78%. The assessment was used to summarize reporting completeness, methodological transparency, and reproducibility and was not intended as a structured risk of bias assessment. The item-specific mean scores were used to identify common strengths and limitations in the reporting of the included studies.

The highest mean item score was observed for clearly defined research questions (Q1: 1.93), indicating that most studies stated their aims adequately. Participant demographics were also generally reported with moderate clarity (Q3: 1.44), whereas inclusion and exclusion criteria were less consistently described (Q4: 1.14). Reporting of familiarization or practice procedures was limited overall (Q5: 1.00), and the replicability of the experimental protocols showed moderate reporting quality (Q6: 1.23). In contrast, sample-size justification was rarely provided, as reflected by the low score for power analysis (Q2: 0.40).

Items directly related to the assessment of running stability showed moderate reporting quality for the methods used to quantify stability (Q7: 1.53) and for the data used for stability assessment (Q8: 1.49). However, test–retest reliability was rarely performed or appropriately referenced (Q9: 0.09), representing the lowest-scoring item. Items Q7–Q9 were particularly relevant to the present methodological review, as they directly addressed the transparency and reproducibility of the stability assessment itself. Thus, although many studies provided sufficient information to understand the applied stability methods and input data, evidence regarding the reliability of these measures remains limited.

## Discussion

This systematic methodological review aimed to identify, evaluate, and synthesize the quantitative methods used to assess stability during running, and to clarify the stability constructs represented by these methods. The main finding was that running stability has been operationalized using a heterogeneous range of approaches, which could be broadly grouped into linear methods, nonlinear methods, and coordination-based methods. Linear methods were mainly used to quantify the magnitude of movement variability or discrete features of movement execution, whereas nonlinear methods primarily addressed the temporal structure of movement variability. Coordination-based methods, in contrast, examined how variability among elemental variables is organized to stabilize task-relevant performance variables, thereby addressing the functional role of movement variability. Across the included studies, nonlinear methods, particularly maximum Lyapunov exponent analyses, were most frequently applied, whereas coordination-based methods were less common but provided a distinct functional perspective on task stability. Overall, the findings indicate that running stability is not assessed by a single interchangeable method, but rather by methods that reflect different theoretical assumptions, input variables, and methodological requirements. Therefore, the choice of stability method should be guided primarily by the research question and the stability construct of interest, while also considering the characteristics of the available biomechanical data, methodological requirements, and practical constraints of the experimental design.

### Linear methods

Linear methods represented a heterogeneous group of approaches and were applied to different stability-related constructs. Although most studies in this category used the term dynamic stability [19, 64, 66–72], others focused on more specific constructs such as rearfoot stability [58, 59], ankle stability [60, 65], body stability [61], locomotor stability [62], or stability of coordination patterns [63]. The methods therefore ranged from dispersion-based measures, such as standard deviation [61, 63] and RMS-based variables [19, 62, 66, 70, 71], to discrete kinematic or kinetic features, including peak values [58–60, 64, 65, 67, 68], range of motion [58, 59, 68], displacement-based measures [68, 72], and margin of stability [69]. Accordingly, these measures should not be interpreted as interchangeable indicators of a single stability construct [15, 24, 57, 63]. Rather, they describe different biomechanical features that may be related to stability in specific contexts, such as frontal-plane rearfoot or ankle motion, trunk movement variability, or center of pressure displacement.

An important distinction is that most linear approaches primarily quantify the amount of variability or the magnitude of discrete biomechanical excursions, while providing limited information about the temporal structure of variability [27, 92]. Measures such as standard deviation, coefficient of variation, range of motion, or peak-based variables can indicate whether movement fluctuations or excursions are larger or smaller, but they do not capture stride-to-stride dependence, predictability, or how deviations evolve across consecutive strides [27, 93, 94]. This distinction is important because variability and stability are related but not equivalent, and greater variability should not automatically be interpreted as reduced stability [24, 27, 34–36, 95]. Rather, variability may also reflect adaptability, flexibility, or task-specific exploration in response to changing constraints [37–39]. Thus, linear methods provide useful descriptive information about movement consistency and discrete features of movement execution, such as peak values, or ranges of motion. When interpreted relative to predefined stability boundaries or task-specific limits, they may also indicate how close movement patterns are to potentially unstable regions [96, 97]. However, in the absence of such reference limits, these measures should be interpreted cautiously, because they do not by themselves describe the temporal structure of variability or how deviations evolve across consecutive strides.

A further limitation concerns the number of analyzed strides, particularly for linear variability measures. In the present review, only five linear-method studies reported the number of cycles analyzed, which ranged from 4 to 20 strides [19, 60, 62, 70, 71]; the remaining studies did not specify this information (Table 4). This is relevant because many linear variability outcomes may be sensitive to the number of strides included in the analysis [93, 94, 98]. Riva et al. [93] showed in walking that the number of strides required for reliable variability and stability estimates was often larger than conventionally used, with standard deviation and coefficient of variation requiring 125 and 127 strides, respectively, before the estimates reached a stable value, defined as an interquartile range/median ratio below 10%. More directly for running, Godin et al. [94] reported that linear variability measures of spatiotemporal parameters required between 16 and 400 steps for representative values and generally showed questionable between-session reliability. Therefore, the low number of analyzed strides in several linear-method studies may particularly limit the reliability of variability-based estimates, whereas discrete peak- or range of motion-based measures may be less directly affected by the number of strides but still require clear reporting and reliability justification. This issue is especially relevant for overground running studies using optical motion capture, where the capture volume often restricts the number of consecutive strides that can be recorded. Although multiple short trials can be combined, this may introduce additional variability related to approach-speed variation, targeting of force plates, or trial-to-trial differences in running execution [13, 99, 100]. Treadmill running allows longer continuous recordings, but may also impose task-specific constraints [101, 102]. Thus, researchers using linear variability measures should justify the number of analyzed strides and, where possible, report reliability or minimal detectable change.

Finally, caution is required when transferring stability measures from walking to running. For example, one included study used the margin of stability [69], which is based on the relationship between the extrapolated center of mass and the base of support [103]. This concept is closely linked to inverted-pendulum models of walking [50]. Running, however, includes a flight phase, lacks double support, and is more appropriately represented by spring-mass dynamics, in which stability emerges from the interaction between leg stiffness, body dynamics, and ground contact mechanics [46, 51, 53, 104]. Therefore, walking-derived measures such as the margin of stability may not be directly applicable to running unless their mechanical assumptions are explicitly justified.

### Nonlinear methods

 Stergiou and Decker [27] in contrast to linear methods, which mainly quantify the amount of variability, nonlinear methods examine how fluctuations are organized over time [27, 95, 105]. In the included studies, maximum Lyapunov exponent analysis was the most frequently used nonlinear method and was mainly interpreted as a measure of local dynamic stability (Table 5). Other nonlinear methods were less common and included sample entropy [19, 66, 70, 71, 73], detrended fluctuation analysis [60, 78], Poincaré maps [75], and maximum Floquet multipliers [80].

This imbalance is notable because orbital stability, commonly quantified using maximum Floquet multipliers, has been more extensively discussed in the broader walking literature as a method for assessing whether a periodic movement pattern returns to its limit cycle after small perturbations [29]. In the present review, however, only two studies used Poincaré map-based approaches, including maximum Floquet multipliers, to assess local orbital stability during running [75, 80]. Local orbital stability captures a different aspect of stability than local dynamic stability [28, 95]. Specifically, local orbital stability assesses whether deviations from a periodic movement pattern decrease discretely from one stride to the next, indicating a return toward the underlying limit cycle [28, 29, 95]. In practical terms, this reflects whether a deviation occurring at one point in the stride cycle, such as at foot contact, is damped or carried over into subsequent strides. In contrast, local dynamic stability quantifies the average exponential rate of divergence of neighbouring movement trajectories in state space [34, 63]. Practically, local dynamic stability indicates how small movement deviations are amplified or attenuated within the movement trajectory, reflecting the system’s sensitivity to small perturbations. Thus, these methods provide complementary information regarding stride-to-stride recovery behavior and local sensitivity to perturbations.

Similarly, detrended fluctuation analysis was applied only in two studies [60, 78]. Although detrended fluctuation analysis was interpreted in relation to global stability in these studies, this interpretation should be made with caution. Detrended fluctuation analysis scaling exponent (DFA-α) primarily quantifies the temporal correlation structure of stride-to-stride fluctuations [106, 107]. In terms of stride pattern, this describes whether deviations in stride variables tend to persist or are corrected across subsequent strides. For example, persistent fluctuations indicate that a longer-than-average stride time is more likely to be followed by another longer-than-average stride time, whereas anti-persistent fluctuations indicate alternating corrections, such that a longer-than-average stride time is more likely to be followed by a shorter-than-average stride time [107, 108]. Values between 0.5 and 1 indicate persistent long-range correlations [106, 107] and may be interpreted in relation to detrended fluctuation analysis-based global stability. However, this interpretation is limited to this range. Values below 0.5 indicate anti-persistent dynamics, whereas values above 1 may reflect nonstationary or Brownian-like dynamics rather than physiological long-range power-law correlations [106, 107, 109]. Thus, detrended fluctuation analysis provides useful information about the temporal organization of running variability, but detrended fluctuation analysis-based global stability should be clearly distinguished from maximum Lyapunov exponent-based local dynamic stability and Poincaré map-based orbital stability, because these methods quantify different properties of movement dynamics [28, 29, 63].

Another methodological issue concerns the choice of input signals. Several maximum Lyapunov exponent studies reconstructed the state space from vertical movement signals [14, 18, 60, 63, 81]. The use of vertical movement may provide a more consistently cyclic input signal for state-space reconstruction and may reduce nonstationarity related to anterior–posterior position changes on the treadmill or treadmill-belt regulation [14, 74]. However, restricting the analysis to the vertical component also excludes potentially relevant information from the anterior–posterior and mediolateral directions. These directions may reflect direction-specific aspects of running, including braking-propulsion dynamics and side-to-side balance control [104, 110, 111]. Therefore, although vertical signals may facilitate state-space reconstruction, they provide only a reduced representation of the multidirectional dynamics of running.

Most studies applying maximum Lyapunov exponent reconstructed the state space from one recorded signal and applied nonlinear analyses directly to measured time series, such as marker trajectories, segment angular velocities, or accelerations (Table 5). Some studies used unfiltered signals [13, 76, 80, 86, 87], whereas others applied filtering to reduce high-frequency measurement artefacts [14, 17, 18, 60, 61, 63, 73, 77, 79, 81–84]. Using relatively direct input signals may reduce dependence on additional preprocessing and modelling choices, which is relevant because maximum Lyapunov exponent estimates are sensitive to filtering, state-space reconstruction parameters, and the selected input signal [95, 112, 113]. At the same time, single-signal reconstructions may not fully represent the multi-segmental nature of running. Only a few studies used more complex input structures. For example, von Diecken et al. [20] combined multiple IMU signals into a 21-dimensional state space, and Walsh [83] applied maximum Lyapunov exponent analysis not only to trunk acceleration but also to muscle synergies. Such approaches may provide a more comprehensive representation of running dynamics, but they also increase methodological complexity and require careful justification of signal selection, dimensionality, and preprocessing.

Finally, the number of analyzed cycles remains an important concern for nonlinear methods. Most studies using maximum Lyapunov exponent analysis analyzed approximately 100 strides (Table 6), which may be sufficient for short-term maximum Lyapunov exponent estimates. However, 100 strides may be insufficient for long-term Lyapunov exponent estimates, orbital stability measures, or other nonlinear methods requiring longer time series [29, 93, 94]. Riva et al. [93] showed in walking that long-term Lyapunov exponents required at least 110 strides before the estimates reached even a broad steadiness criterion, defined as an interquartile range/median ratio below 50%, and were classified as having very poor within-session reliability. More recent running-specific evidence also indicates that nonlinear variability measures of spatiotemporal variables may require several hundred steps for representative estimates [94]. Therefore, nonlinear analyses should not rely on a fixed convention of 100 strides without considering the specific method, input signal, and stability construct. Future studies should report the number of analyzed cycles, justify whether the time-series length is adequate for the chosen method, and clearly distinguish between short-term and long-term local dynamic stability if Lyapunov exponent measures are applied.

### Coordination-based methods

The included studies mainly interpreted stability as performance-variable stability [21–23, 88–90], whereas one study used the term system stability [91]. Elemental variables included lower-body joint angles [22, 88, 91], full-body joint angles [21–23, 90], or leg length and leg angle [89]. Performance variables included center of mass position in two [22, 89] or three dimensions [21–23, 90], vertical foot position [88], or horizontal and vertical leg position [91]. Although these choices can be justified from a biomechanical perspective, the interpretation of uncontrolled manifold outcomes depends strongly on which elemental and performance variables are selected [31, 95, 118]. A performance variable may be biomechanically relevant, but this does not necessarily prove that it is explicitly stabilized or prioritized by the nervous system during running [95, 118].

A further conceptual limitation concerns the coordinate dependence of covariance-based variability analyses [118]. The uncontrolled manifold approach relies on decomposing variability into directions parallel and orthogonal to the null space of the Jacobian [26]. However, orthogonality and anisotropy of a covariance matrix are sensitive to the coordinate system in which the data are represented [118]. Consequently, a change in coordinate definition or scaling can influence the distribution of variability and therefore the interpretation of whether variability is aligned with the uncontrolled manifold [118]. This does not invalidate the uncontrolled manifold approach, but it means that the results should be interpreted as evidence of stabilization within the selected task model and coordinate system, rather than as coordinate-independent proof of a specific motor control strategy.

This issue is particularly relevant for running because the included studies differed in model dimensionality and variable choices (Table 8). Full-body three-dimensional models incorporate contributions from more segments and planes of movement, but they also require additional modelling choices and increase computational and interpretative complexity [21–23, 90]. Simpler two-dimensional or lower-body models may be easier to interpret [89, 91], but may omit relevant contributions from other segments, planes of motion, or upper-body coordination. Similarly, different performance variables may lead to different conclusions about which aspect of running is stabilized. Therefore, future studies should clearly justify why the selected performance variable is task-relevant and likely to be stabilized by the central nervous system, and should report coordinate definitions, model assumptions, and the mathematical mapping between elemental and performance variables in sufficient detail.

### Important considerations when choosing stability methods

When interpreting running stability methods, it is important to distinguish between stability, robustness, variability, and consistency. Although robustness was not frequently addressed explicitly in the included studies, it is closely related to stability and is often used interchangeably or imprecisely in the broader literature. In running, stability can be broadly defined as the ability of the motor control system to return to a steady-state running pattern after a perturbation [24, 119]. Robustness is related but not identical; it describes the ability to withstand internal or external perturbations and maintain the running pattern without substantial deviations in movement strategy [95, 120]. Variability, in contrast, refers to fluctuations across repeated executions of a task, such as stride-to-stride differences [27]. The amount of variability, as quantified by many linear methods, can provide useful information about movement consistency, but it should not be equated directly with stability. Greater variability should therefore not automatically be interpreted as lower stability, because variability may reflect not only deviations from the intended movement pattern, but also functional flexibility in adapting to task demands [27, 32, 35, 121]. Therefore, reduced variability or higher consistency should be interpreted as improved stability only with caution.

Nonlinear methods address a different aspect by quantifying the temporal structure of variability and, for methods such as maximum Lyapunov exponents or Floquet multipliers, how deviations evolve over time [25, 29]. These methods are therefore more closely linked to stability definitions based on divergence, recovery, or return behavior than linear variability measures, although their interpretation still depends on the specific nonlinear method applied. Coordination-based methods provide yet another perspective by evaluating whether variability among elemental variables is organized to stabilize a selected performance variable [26, 31]. However, these interpretations also depend on the chosen coordinate system, elemental variables, and performance variable.

A second important consideration is methodological transparency. Across all method categories, interpretation depends strongly on processing decisions, although the relevant details can differ between methods. For measures based on repeated cycles, the number of analyzed strides or steps should be reported and justified [93, 94, 122, 123]. For nonlinear analyses, filtering [113], normalization procedures [113, 124], and method-specific parameters should be described in detail, including state-space reconstruction parameters for Lyapunov exponent analyses [95, 112, 113], embedding dimension and tolerance radius for sample entropy analyses [42, 125], and box-size range, increment method, and detrending order for detrended fluctuation analysis [109]. For coordination-based methods, model assumptions [26, 118], coordinate definitions [118], and the mapping between elemental and performance variables [26, 43, 118] should be clearly specified. These details are not only relevant for reproducibility, but can also affect the resulting stability estimates and their interpretation. Future studies should therefore report these methodological choices in sufficient detail and justify them in relation to the selected stability construct.

The quality assessment further supports the need for greater methodological transparency (Supplementary Table 4). Although the quality assessment was not used to exclude studies, it showed that reporting completeness varied across the included literature. In particular, several studies did not provide sufficient detail on sample-size justification, familiarization procedures, number of analyzed strides, processing decisions, or reliability of the applied stability measures. These limitations do not necessarily invalidate the individual findings, but they restrict reproducibility and make comparisons across studies more difficult. Therefore, future running stability studies should report not only the selected stability outcome, but also the methodological decisions that directly affect its interpretation.

Finally, the limits of the chosen method should explicitly be acknowledged. Linear measures mainly quantify the magnitude of variability [27] or discrete features of movement execution, such as peak values, or range of motion [58, 60, 68]. Nonlinear methods describe different properties of movement dynamics, including temporal structure and predictability [27, 95, 109], divergence behavior in Lyapunov exponent analyses [25, 95], or return behavior in orbital stability analyses [29]. Coordination-based methods evaluate whether variability among elemental variables is functionally organized with respect to a selected performance variable [26, 31], but their interpretation depends on the selected variables, model, and coordinate system [118]. None of these methods provides a complete or universal measure of running stability. Thus, the method should be selected according to the specific research question, and conclusions should be restricted to the stability construct that the chosen method quantifies.

## Limitations and future directions

This review has several limitations. First, the included studies were heterogeneous with respect to experimental tasks, participant groups, running conditions, measurement systems, input variables, and stability metrics (Supplementary Tables 1–3). Although this heterogeneity reflects the current diversity of running stability research, it limited quantitative synthesis and direct comparison of effect sizes across methods. Second, the classification into linear, nonlinear, and coordination-based methods was used as a broad conceptual framework for the synthesis. Although these categories represent distinct methodological approaches that quantify different properties of movement, grouping heterogeneous methods into three broad categories inevitably simplifies some method-specific differences. For example, nonlinear methods included approaches that assess local divergence, signal regularity, or long-range correlations, whereas linear methods included both dispersion-based measures and discrete features of movement execution. Thus, the classification should be understood as a framework for synthesizing the literature, rather than as a complete representation of all methodological distinctions among individual measures. Third, the quality assessment focused on methodological transparency and reporting completeness rather than risk of bias or study validity. Therefore, the quality scores should be interpreted as indicators of reporting quality, not as direct measures of scientific quality. Fourth, the exclusion of records containing *gait* in the title represents a potential limitation, as this term is also used in running research. To assess the potential impact of this restriction, a complementary sensitivity search was conducted on 28.08.2026 using the same search strategy but requiring *gait* in the title instead of excluding it. This search identified 138 records after duplicate removal, but none met the eligibility criteria, suggesting that this restriction did not affect the final study selection. Finally, because the review focused on published English-language studies, relevant work outside these criteria may have been missed.

Future research should move toward more standardized reporting and clearer construct definitions in running stability analysis. Studies should justify the selected stability method in relation to the research question, report key processing parameters in sufficient detail, and provide reliability or sensitivity analyses where possible. Given the increasing use of IMUs and other wearable sensors in applied running research, future studies should consider how measurement and processing choices, including sensor placement, sampling frequency, and signal-processing procedures, may influence stability estimates and comparability across measurement systems. More work is also needed to determine how different methods respond to the same running conditions, perturbations, or task constraints. In particular, future studies should compare linear, nonlinear, and coordination-based outcomes within the same datasets to clarify whether they provide complementary or redundant information. Finally, greater use of perturbation-based running paradigms could help distinguish variability, consistency, robustness, and stability by examining responses to controlled perturbations.

## Conclusion

This systematic methodological review shows that running stability has been assessed using a broad range of quantitative methods that reflect different theoretical assumptions and stability constructs. Linear methods mainly quantified the magnitude of variability or discrete features of movement execution. Nonlinear methods primarily characterized the temporal structure of variability, including trajectory divergence, signal regularity, temporal correlations, and return behavior. Coordination-based methods examined how variability among elemental variables is functionally organized to stabilize a selected performance variable. Because these approaches capture different properties of movement, they are not interchangeable, and the present review does not indicate that any one approach is superior to the others. The findings highlight the need for clearer terminology, method-specific interpretation, and more transparent reporting in running stability research. In particular, future studies should justify the selected method in relation to the research question and report key methodological details such as input variables, number of analyzed strides, filtering, normalization, and model assumptions. Furthermore, stability methods should be selected according to the specific construct of interest, and results should be interpreted within the conceptual and methodological limits of the selected approach.

## Supplementary Information

Supplementary Material 1.

## Data Availability

All data extracted and analyzed in this review are included in this article and its supplementary information files.

## Abbreviations

DFA: Detrended fluctuation analysis
DFA-α: Detrended fluctuation analysis scaling exponent
IMU: Inertial measurement unit
MLE: Maximum Lyapunov exponent
PERSiST: PRISMA in Exercise, Rehabilitation, Sport medicine and SporTs science
PRISMA: Preferred Reporting Items for Systematic Reviews and Meta-Analyses
Q1–Q9: Quality assessment items 1–9
RMS: Root mean square
SDMO: Studies, Data, Methods, Outcomes
UCM: Uncontrolled manifold
UCM_∥_: Variability parallel to the uncontrolled manifold
UCM_⊥_: Variability orthogonal to the uncontrolled manifold
