# Supplementary material for: Assessing running stability: a systematic review of methods
Source: BMC Sports Sci Med Rehabil. 2026 Sep 9;18:414. doi: 10.1186/s13102-026-02062-4 (PMC13570515; doi:10.1186/s13102-026-02062-4)
Supplement: Supplementary file 1 [file 13102_2026_2062_MOESM1_ESM.pdf]

Supplementary Table 1 Sample characteristics of the included studies. The table summarizes whether a power analysis was reported, sample size, number of female participants, age, height, body mass, health status, and training status. Values are presented as mean ± standard deviation. Female participants are indicated in parentheses. Excluded participants are reported separately when available. Group-specific values are provided for studies that reported participant characteristics separately by group.

Study	Power analysis	Size (number of F)	Age (years)	Height (m)	Mass (kg)	Health status	Training status
Agresta et al., 2019 [78]	No	38; 2 excluded	38.6±10.4	Novice: 65.7 ± 5.7; Intermediate: 69.3 ± 3.6; Experienced: 70.1 ± 4.0	Novice: 66.4 ± 10.2; Intermediate: 70.7 ± 11.7; Experienced: 75.2 ± 11.3	Healthy	Novice: ≤3 years; Intermediate: 4-9: Experienced: ≥10 years of distance running experience
Aristizábal Pla et al., 2021 [66]	No	132 (42 F); 76 excluded	18.7 ± 2.0	1.76 ± 0.08	67.8 ± 9.1	Healthy	Following an academic sport program, 10 h/week (various sports types)
Arshi et al., 2015 [73]	No	15 (0 F)	24.1 ± 1.0	1.76 ± 0.04	68.8 ± 3.8	Healthy	NS
Ataabadi et al., 2021* [61]	Yes	23 (13 F)	22.04 ± 3.43	1.68 ± 0.07	62.14 ± 9.26	Healthy	active
Blum et al., 2011 [75]	No	7 (1 F)	NS	NS	77 ± 9	NS	NS
Cerrito et al., 2025 [87]	Yes	26 (12 F), 10 excluded	30.9 ± 3.5	1.76 ± 0.09	68.9 ± 11.8	Healthy	Novice: ≤6 months; Expert ≥4 years of distance running experience
Dias et al., 2025 [88]	No	28 (1 F)	34.75 ± 6.7	1.76 ± 0.07	69.6 ± 7.7	No injuries that could affect their running style	Experienced runners (101 ± 84 months of practice)
Ekizos et al., 2017 [14]	No	20 (6 F)	27.8 ± 5	1.79 ± 0.08	72.9 ± 11.9	Healthy	Walked and ran habitually shod in their daily life
Ekizos et al., 2018 [81]	No	16 (5 F)	27 ± 5	1.79 ± 0.09	72 ± 12	Healthy	NS
Fohrmann et al., 2023 [13]	No	25 (12 F)	34.1 ± 10.0	1.75 ± 0.08	69.4 ± 12.4	Healthy	NS

Fohrmann et al., 2025 [76]	Yes	26 (15 F), 9 excluded	33.6 ± 9.8	1.78 ± 0.06	74.7 ± 8.9	Healthy	NS
Frank et al., 2019 [17]	No	24 (0 F)	Novice: 21.5 ± 2.7; Trained: 23.3 ± 5.8	Novice: 1.77 ± 0.06; Trained: 1.75 ± 0.05	Novice: 75.8 ± 9.8; Trained: 68.3 ± 5.1	Healthy	Novice: less than 10 km in the past year; Trained: 61.7 km/week
Garofolini et al., 2024 [91]	No	20 (0 F)	31.2 ± 6.9	1.77 ± 0.07	73.4 ± 7.9	Healthy	Running for ≥5 years, ≥40 km/week
Hagen et al., 2025 [58]	Yes	20 (0 F)	26.9 ± 2.4	1.84 ± 0.08	73.3 ± 6.2	Healthy	NS
Hilário et al., 2022 [69]	No	12 (12 F), 8 excluded	NS	only BMI	only BMI	Healthy	Training 5.12 ± 3.13 years, 25.16 ± 7.15 km/week
Hoenig et al., 2019 [86]	No	30 (0 F)	Recreational: 25.3 ± 7.6; Competitive: 28.7 ± 4.3	only BMI	only BMI	Healthy	Recreational: 15.9 ± 10.0 km/week; Competitive: 53.2 ± 26.9 km/week
Hollander et al., 2021 [84]	No	37 (before dropout 21 F), 4 excluded	Before dropout: Barefoot group: 25.2 ± 3.4; Footwear intervention group: 25.2 ± 2.9	Before dropout: Barefoot group: 1.75 ± 0.08; Footwear intervention group: 1.78 ± 0.08	Before dropout: Barefoot group: 71.3 ± 12.2; Footwear intervention group: 71.4 ± 10.8	Healthy	NS
Iosa et al., 2013 [62]	No	40 (16 F)	Cerebral palsy: 5.1 ± 2.3; Typical development: 5.9 ± 2.7	Cerebral palsy: 1.10 ± 0.17; Typical development: 1.20 ± 0.20	NS	Cerebral palsy and healthy	NS
Jordan et al., 2009 [63]	No	12 (12 F)	26.2 ± 2.9	1.67 ± 0.04	62.4 ± 5.6	Healthy	Recreational runners
Kelly et al., 2022 [64]	Yes	30 (16 F)	Plantar fasciitis: 43 ± 11; Resolved plantar fasciitis: 43 ± 13; Control: 38 ± 11	Plantar fasciitis: 1.65 ± 0.10; Resolved plantar fasciitis: 1.70 ± 0.08; Control: 1.65 ± 0.08	Plantar fasciitis: 67.7 ± 12.9; Resolved plantar fasciitis: 71.0 ± 15.1; Control: 65.0 ± 10.5	Plantar fasciitis and healthy	Plantar fasciitis: ≥9.65 km/week; Resolved plantar fasciitis and Control: ≥24.14 km/week

Kettner et al., 2025a [60]	No	17 (0 F)	25.7 ± 3.9	1.78 ± 0.04	68.1 ± 6.0	Healthy	4.2 ± 1.8 days/week and 33.7 ± 22.4 km/week
Kettner et al., 2025b [21]	No	17 (0 F)	25.7 ± 3.9	1.78 ± 0.04	68.1 ± 6.0	Healthy	4.2 ± 1.8 days/week and 33.7 ± 22.4 km/week
Law et al., 2018 [70]	No	20 (0 F)	22.5 ± 2.6	1.7 ± 0.2	76.5 ± 22.4	Healthy	NS
Liew et al., 2024 [89]	No	28 (1 F)	34.8 ± 6.7	1.76 ± 0.07	69.6 ± 7.7	Healthy	16–25 km [n = 1], 26–35 km [n = 9], 36–45 km [n = 7], 45 km [n = 2], > 45 km [n = 9];
Look et al., 2013 [74]	No	17 (5 F)	NS	NS	NS	Leg amputation and healthy	NS
Mehdizadeh et al., 2014 [85]	No	15 (0 F)	24.1 ± 1.0	1.76 ± 0.04	68.8 ± 3.9	Healthy	NS
Mehdizadeh et al., 2016 [80]	No	10 (0 F)	23.3 ± 2.9	1.79 ± 0.04	71.6 ± 4.8	Healthy	8.8 ± 3.2 years football experience at national level
Möhler et al., 2019 [22]	No	13 (0 F)	23.5 ± 3.6	1.80 ± 0.06	66.8 ± 5.4	Healthy	7.2 ± 3.2 years of running experience, 6.5 ± 1.7 h/week running
Möhler et al., 2020 [23]	No	25 (0 F)	Experts: 23.5 ± 3.6; Novices: 23.9 ± 3.8	Experts: 1.80 ± 0.06; Novices: 1.83 ± 0.07	Experts: 66.8 ± 5.4; Novices: 72.2 ± 6.6	Healthy	Experts:7.2 ± 3.2 years of running experience, 6.5 ± 1.7 h/week running; Novices: no running experience, 0.2 ± 0.2 h/week running
Möhler et al., 2022 [90]	No	14 (0 F)	27.4 ± 4.3	1.82 ± 0.06	77.5 ± 10.3	Healthy	14 ± 18 min/week running
Ogaya et al., 2024 [79]	No	18 (18 F)	20.1 ± 2.6	159.6 ± 5.6	56.0 ± 5.5	Healthy	NS
Promsri, 2022 [77]	Yes	22 (12 F)	25.7 ± 3.3	1.75 ± 0.08	67.9 ± 9.3	Healthy	5-10 h/week (13.6%), 1-5 h/week (72.7%), 0 h/week (13.6%)
Rethwilm et al., 2021 [71]	No	117 (50 F)	11.0 ± 3.2	Cerebral palsy: 1.45 ± 0.17; Typical development: 1.45 ± 0.16	Cerebral palsy: 37.7 ± 12.9; Typical development: 36.8 ± 12.4	Cerebral palsy and healthy	NS

Santuz et al., 2018 [18]	No	18 (7 F)	24 ± 3	1.76 ± 0.07	71 ± 13	Healthy	NS
Schütte et al., 2016 [68]	No	30 (14 F)	22.62 ± 3.07	1.76 ± 6.24	63.05 ± 5.57	Healthy	Recreational: 9.56 ± 11.88 km/week; Highly trained: 72.88 ± 7.94 km/week
Schütte, Sackey, et al., 2018 [67]	No	30 (14 F)	21.75 ± 1.40	1.73 ± 0.08	68.18 ± 11.41	Healthy	Inclusion: 2–4 sessions per week; 15–40 km/week
Schütte, Seerden, et al., 2018 [19]	Yes	30 (12 F)	Medial tibial stress syndrome: 20.36 ± 0.84; Control: 20.13 ± 0.72	Medial tibial stress syndrome: 1.77 ± 0.10; Control: 1.75 ± 0.07	Medial tibial stress syndrome: 68.29 ± 9.02; Control: 63.06 ± 9.45	Medial tibial stress syndrome and healthy	Medial tibial stress syndrome: 3.15 ± 1.33 years of running experience, 24.14 ± 5.64 km/week; Control: 3.22 ± 1.57 years of running experience, 26.44 ± 6.26 km/week
Sterzing et al., 2015 [59]	No	24 (0 F)	20.9 ± 1.9	1.79 ± 0.07	70.4 ± 6.3	Healthy	6.0 ± 2.4 years of running experience, 15.0 ± 7.8 km/week
von Diecken et al., 2025 [20]	No	21 (9 F)	Male: 34.9 ± 8.3; Female: 29.1 ± 10.2	BMI and mass stated	Male: 71.1 ± 8.5; Female: 62.3 ± 5.9	Healthy	32.4 ± 12.0 km/week
Walsh, 2021 [83]	No	18 (9 F)	Male: 31 ± 4; Female: 27 ± 9	Male: 1.80 ± 0.08; Female: 1.70 ± 0.11	Male: 77.5 ± 7.9; Female: 69.4 ± 15.0	Healthy	NS
Wang & Yuan, 2024 [65]	No	30 (17 F)	Ankle brace: 23.84 ± 2.56; Control: 23.25 ± 2.39	only BMI	only BMI	Healthy	Ankle brace: 8.17 ± 4.96 h/week training; Control: 9.21±5.15 h/week training
Xiang et al., 2022 [82]	No	25 (0 F)	24.5 ± 3.0	1.74 ± 0.06	68.6 ± 8.2	Healthy	Inclusion: ≥20 km/week
Zhang et al., 2024 [72]	Yes	120 (0 F), 3 excluded	No pain: 21.3 ± 1.1; Anterolateral: 22.2 ± 1.8; Mediolateral: 23.1 ± 2.5;	No pain: 1.79 ± 0.05; Anterolateral: 1.80 ± 0.07; Mediolateral: 1.77 ± 0.06;	No pain: 67.4 ± 6.5; Anterolateral: 68.1 ± 5.9; Mediolateral: 67.9 ± 6.5;	Chronic ankle instability and healthy	NS

			Posterolateral: 21.8 ± 2.1	Posterolateral: 1.79 ± 0.05	Posterolateral: 69.3 ± 5.1		

Abbreviations: BMI, body mass index; F, female; h/week, hours per week; kg, kilogram; km/week, kilometers per week; m, meter; min/week, minutes per week; n, number of participants; NS, not stated; SD, standard deviation.

Supplementary Table 2 Experimental running protocols and analytical approaches of the included studies. The table summarizes the running environment, warm-up or familiarization procedures, main running task, whether reliability and validity were assessed, and the statistical analyses used. Running speed, duration, distance, number of trials, and task conditions are reported as described in the original studies.

Study	Treadmill/ Overground	Warmup	Running task	Reliability	Validity	Statistics
Agresta et al., 2019 [78]	Treadmill	Run with self-selected speed	5 min baseline run; 5 perturbations with metronome where participants must match footfalls to beats; 3 min run in silence before test ended	No	No	Linear regression; GLMM
Aristizábal Pla et al., 2021 [66]	Overground	800 m run with self-selected speed and drills	12 min run as fast as possible	No	No	mixed ANOVA
Arshi et al., 2015 [73]	Treadmill	Run with self-selected speed	2 min in forward and lateral running directions	No	No	rmANOVA
Ataabadi et al., 2021* [61]	Treadmill	Run on 10-m pathway at their preferred speed six times	Run at preferred speed, 20% slower and faster than preferred speed, 2 min each	No	No	rmANOVA
Blum et al., 2011 [75]	NS	NS	Run at 2 m/s, 3 m/s & 4 m/s	No	No	NS
Cerrito et al., 2025 [87]	Treadmill	Squats-to-calf raises, squat-lunges, and two running drills, running on treadmill	Run at preferred speed, 10% slower and faster than preferred speed, 10 min each	No	No	rmANOVA
Dias et al., 2025 [88]	Treadmill	1 min walking at 1.2 m/s	3 min 30 s run at 2.5, 3.5, and 4.5 m/s	No	No	rmANOVA
Ekizos et al., 2017 [14]	Treadmill	40-60 s familiarization before each condition	2 min shod and barefoot run at self-selected speed	No	No	t-test
Ekizos et al., 2018 [81]	Treadmill	40-60 s familiarization before each condition	2 min run at self-selected speed; repeated on six different days	Yes	No	rmANOVA, ICC
Fohrmann et al., 2023 [13]	Treadmill and overground	5 min run at self-paced speed, 300 m familiarization before overground trials	5 min treadmill run at self-paced speed; 10 laps across 50 m path with indoor and outdoor overgrounds; repeated three times on two days	Yes	No	rmANOVA, ICC

Fohrmann et al., 2025 [76]	Treadmill	5 min familiarization 10 km/h and 5 min warmup at 10 km/h (for 1 min at each of five body weight support levels	3 min run at 12 km/h with body weight support levels randomized between 0% and 80% in 10% increments	No	No	LMM
Frank et al., 2019 [17]	Treadmill	Run until they reached an RPE score equal to a moderate effort	4 min run at preferred running speed for each shoe condition	No	No	LMM
Garofolini et al., 2024 [91]	Treadmill	7 min progressive warm-up	5 min run at 11 km/h for each shoe condition	No	No	rmANOVA
Hagen et al., 2025 [58]	Overground	NS	5 trials at 3.3 m/s ± 3% per leg	No	No	MANOVA
Hilário et al., 2022 [69]	Treadmill	3 min at self-selected speed	3 x 1 min at self-selected speed	No	No	rmANOVA
Hoenig et al., 2019 [86]	Overground	1,200 m easy run	5,000 m as fast as possible	No	No	rmANOVA
Hollander et al., 2021 [84]	Treadmill	NS	15 min at 70% of their individual VO2max velocity	No	No	LMM
Iosa et al., 2013 [62]	Overground	NS	10 trials at self-selected speed	No	No	mixed ANOVA
Jordan et al., 2009 [63]	Treadmill	45 min treadmill on a separate day for familiarization, 5-10 min warm-up at self-selected speed	Walk and run for 5 min at speeds associated with preferred gait transition speeds.	No	No	rmANOVA
Kelly et al., 2022 [64]	Overground	NS	Run at 3.3 m/s ± 5%	No	No	ANOVA
Kettner et al., 2025a [60]	Treadmill	5 min self-selected speed	90 s at 10 km/h and 15 km/h	No	No	SPM
Kettner et al., 2025b [21]	Treadmill	5 min self-selected speed	90 s at 10 km/h and 15 km/h	No	No	ANOVA
Law et al., 2018 [70]	Overground	Stretching warm-up protocol	5 trials on 15 m pathway	No	No	rmANOVA
Liew et al., 2024 [89]	Treadmill	NS	30 s at 2.5 m/s, 3.5 m/s, and 4.5 m/s	No	No	pairwise marginal difference
Look et al., 2013 [74]	Treadmill	Brief warm-up	3 m/s and increased until individual top-speed is reached; 8 strides for top-speed, 10 strides for slower trials	No	No	NS

Mehdizadeh et al., 2014 [85]	Treadmill	Enough time to familiarize	2 min at 80, 100, and 120% of preferred speed forwards and backwards run	No	No	rmANOVA
Mehdizadeh et al., 2016 [80]	Treadmill	Enough time to familiarize	2 min at 80, 100, and 120% of preferred speed forwards, backwards and lateral run	No	No	rmANOVA
Möhler et al., 2019 [22]	Treadmill	6 min walk, 6 min run	Run until fatigue at their individual fatigue speed	No	No	rmANOVA
Möhler et al., 2020 [23]	Treadmill	6 min walk, 6 min run, 15 km/h for 15 s	1 min at 10 km/h and 15 km/h	No	No	rmANOVA
Möhler et al., 2022 [90]	Treadmill	6 min at 5 km/h, 6 min at 8 km/h, 10 s at 13 km/h	Run until fatigue at 13 km/h	No	No	SPM
Ogaya et al., 2024 [79]	Treadmill	4 min at 2 m/s	30 s at 2 m/s	No	No	t-test
Promsri, 2022 [77]	Treadmill	5 min walk, 5 min run at 10.4 ± 1.0 km/h	30 s at 10.4 ± 1.0 km/h	No	No	t-test
Rethwilm et al., 2021 [71]	Overground	NS	Cerebral palsy: 13 m run at 2.32 ± 0.41 m/s; Typical development: 13 m run at 2.70 ± 0.34 m/s	No	No	rmANOVA
Santuz et al., 2018 [18]	Treadmill	Maximum 60 s accommodation period	2 x 2 min run at 2 m/s for female, 2.2 m/s for male	No	No	rmANOVA
Schütte et al., 2016 [68]	Overground	Standardized warmup	90 m at self-selected speed	No	No	Linear regression
Schütte, Sackey, et al., 2018 [67]	Treadmill	4 min at 2.22-2.5 m/s	2.22-2.5 m/s is increased by 0.42 m/s every 4 min interspersed by a 1-min rest until volitional exhaustion	No	No	Multiple regression
Schütte, Seerden, et al., 2018 [19]	Overground	400 m run with comfortable speed	3200 m run with maximal effort	No	No	Linear regression
Sterzing et al., 2015 [59]	Overground	400 m run	5 x 16 m run at 3.3 m/s ± 3%	No	No	rmANOVA
von Diecken et al., 2025 [20]	Treadmill	NS	5 trials of 3 x 3 min run at 10.31 ± 0.96, 13.43 ± 1.38 and 15.39 ± 1.75 km/h	No	No	LMM
Walsh, 2021 [83]	Treadmill	5 min walk and run at self-selected speed	3 min at self-selected and 120% of self-selected speed under single and dual task conditions	No	No	rmANOVA
Wang & Yuan, 2024 [65]	NS	Warmup	3 x run at 3.0 ± 0.4 m/s	No	No	t-test

Xiang et al., 2022 [82]	Treadmill	10 min	10 km run at 11.2 ± 1.2 km/h	No	No	LMM
Zhang et al., 2024 [72]	Not clear	10 min at 3.5 m/s	5 x 15 m run	No	No	ANOVA

Abbreviations: ANOVA, analysis of variance; GLMM, generalized linear mixed model; ICC, intraclass correlation coefficient; LMM, linear mixed model; MANOVA, multivariate analysis of variance; NS, not stated; rmANOVA, repeated-measures analysis of variance; RPE, rating of perceived exertion; SPM, statistical parametric mapping; VO_2max, maximal oxygen uptake.

Supplementary Table 3 Study aims, main findings, and authors' interpretations of the included studies. The table summarizes the primary research objective of each study, the key stability-related findings, and the practical or clinical interpretations reported by the authors. Findings are presented in relation to the stability construct or methodological approach used in each study.

Study	Aim	Main findings	Interpretation
Agresta et al., 2019 [78]	To examine the extent to which stride-to-stride fluctuations change in response to enforced step frequency perturbations and whether this change was associated with adaptive response	Step frequency perturbations less than preferred were sufficient to alter stride-to-stride fluctuations in step rate but not contact time. More persistent step rate was associated with faster adaptation times across all runners indicating destabilization may be necessary to achieve step frequencies away from preferred.	Baseline locomotor flexibility may be beneficial to achieve task demands and return to a stable state once the task is complete.
Aristizábal Pla et al., 2021 [66]	To investigate accelerometry based measures of dynamic stability, and loading as potential risk factors for the development of lower leg overuse injuries	Participants sustaining an injury within the 6-month period demonstrated higher RMS ratio values in the vertical direction and lower RMS ratio values in the AP direction, and lower impact acceleration values in the AP direction in an unfatigued state compared to the uninjured group.	There is potential for using a single trunk-mounted accelerometer to detect changes in movement dynamics that are linked to lower-leg overuse injuries.
Arshi et al., 2015 [73]	To compare dynamic stability in both the AP and ML directions and to determine whether they differed in forward and lateral running patterns	Lower dynamic stability in the primary plane of progression during both forward and lateral running. Control of stability in AP and ML directions of running is dependent on the direction of progression.	Greater control might be required to regulate system stability in the primary plane of progression during running.
Ataabadi et al., 2021* [61]	To investigate the effects of different running speeds on linear and non-linear lower limb joints angle variability among healthy active runners	Hip and knee joints non-linear variability in the sagittal plane is considerably higher when the running speed increases.	Joints are attempting to maintain body stability by regulating internal body system perturbations by increasing their variability.
Blum et al., 2011 [75]	To evaluate the stability and robustness characteristics of running with a straight versus a crouched leg posture, using both experimental data and predictions of the spring-mass model	Applied control strategies are redundant in stabilizing the spring-mass model.	A crouched leg posture does not necessarily enhance running stability.
Cerrito et al., 2025 [87]	To investigate the effect of the level of experience and running speed on local dynamic stability in the ankle, knee, and hip joints in the sagittal plane	Experienced runners have greater local dynamic stability.	The results should be interpreted with caution, particularly due to the low running speeds chosen by the novice runners.

Dias et al., 2025 [88]	To verify the existence of a multi-joint synergy responsible for stabilizing foot height throughout the entire swing phase during treadmill running	Synergy index was the lowest at the slowest speed in the second half of the swing phase, because of a low $UCM_{parallel}$.	CNS organizes a multi-joint synergy to stabilize foot height, with the strongest synergy at the end of the swing phase to ensure safe foot placement and landing.
Ekizos et al., 2017 [14]	To investigate the local dynamic stability of the human system during an immediate alteration from shod to barefoot running	Local dynamic stability decreased significantly during barefoot running.	Immediate transition from shod to the barefoot condition resulted in an increased instability and indicates poorer control over the movement.
Ekizos et al., 2018 [81]	To examine the reliability of the MLE both during walking and running using six different marker sets fixed on the trunk	All marker-sets showed mostly good ICCs during running.	Assessment of dynamic stability using the MLE is reliable in running.
Fohrmann et al., 2023 [13]	To estimate the test-retest reliability of IMU based measurements of the running stability at different body locations; and to evaluate the agreement of running stability measured on a treadmill, overground in a laboratory-like setting, and overground in an outdoor setting	Sacrum sensor measurements demonstrated the highest reliabilities, while those of the tibia measurements showed the lowest. Treadmill measurements depicted systematically lower values than both overground conditions for all sensor locations.	IMUs show moderate to excellent reliability for both overground and treadmill running.
Fohrmann et al., 2025 [76]	To comprehensively investigate changes in kinematics, kinetics, and running stability during running at different levels of body weight support	With higher body weight support levels, the foot, running stability increased at the tibia running stability decreased.	Running stability showed marginal changes with unloading.
Frank et al., 2019 [17]	To evaluate differences in local dynamic stability between trained and novice runners at a joint level, for the ankle knee and hip	Trained runners had increased movement stability in all shoe conditions compared to their novice counterparts. Midsole thickness and stiffness, overall, did not affect movement stability.	Midsole design within current design ranges do not have the ability to influence movement stability.
Garofolini et al., 2024 [91]	To investigate the consistency of leg length and orientation during the landing phase of running and to understand how different foot landing styles and shoe types might influence this	Forefoot and rearfoot strikes are equally able to reduce task-relevant variance in order to stabilize the control variables leg length and leg orientation. The assistance provided by the shoe may affect the control variables.	There is potential reliance on footwear for stability.
Hagen et al., 2025 [58]	To identify the morphological and biomechanical effects on rearfoot motion during running after two machine-based shank muscle strength training methods	Higher inversion angle at initial contact and reduced eversion velocity in mid-stance after treatment.	Particularly due to the improved stabilization of the rearfoot during running, machine-based muscle strength training of the pronators and supinators

			can be recommended as a preventive measure to prevent overuse injuries in male runners.
Hilário et al., 2022 [69]	To verify whether the use of the infracapital bar of different measures and positions can promote changes in biomechanical variables related to the dynamic stabilization of the lower limbs in recreational runners	Neutral insoles increased the lateral rotation of the left knee and the medial rotation of the right hip. Insoles with a 2 mm medial infra-capital bar increased the adduction of left hip and the medial rotation of right hip. Insoles with a 4 mm medial infra-capital element increased the adduction of left hip, the medial rotation of right hip and the abduction of right knee.	The insoles used modified biomechanical variables of recreational runners related to dynamic stability during running on a treadmill and static baropodometric variables.
Hoenig et al., 2019 [86]	To show the influence of fatigue on local dynamic stability in recreational and competitive runners during track running	Local dynamic stability increased during the run (thorax, pelvis) in both recreational and competitive runners. During the whole run, competitive runners showed a higher MLE compared to recreational runners at the foot kinematics.	The analysis of local dynamic stability during running, quantified by MLE may provide valuable information on sports performance and injury risk.
Hollander et al., 2021 [84]	To determine the effects of repeated test conditions to barefoot running on nonlinear biomechanics, measured as running stability	Participants in the barefoot running group exhibited lower running stability than in the shod running group with no changes during the intervention period. Within a single session, running stability decreased over the course of the 15-min run without differences between both groups.	Changing from shod to barefoot running reduces running stability not only in the acute phase but also in the longer term.
Iosa et al., 2013 [62]	To examine the ability and stability of running in mildly affected children with hemiplegia due to cerebral palsy versus an age-matched group of children with typical development	No changes were observed in terms of gait stability.	During running, children with cerebral palsy are slower than children with typical development, but their gait was not less stable.
Jordan et al., 2009 [63]	To examine the relation between long range correlations of the stride interval measured via DFA, and gait cycle stability as indexed both by the MLE and SD of relative phase	During slow running the local dynamic stability of the head was decreased compared with fast walking, with the reverse being true for the local dynamic stability of the ankle. The standard deviation of relative phase of the knee and ankle also was greater during slow running than fast walking.	Larger long-range correlations in the stride interval are associated with decreases in measures of stability.
Kelly et al., 2022 [64]	To assess dynamic stability, as measured by time-to-contact, in runners with plantar	Time-to-contact values were shorter in plantar fasciitis group compared to the other groups to all boundaries during mid-stance. Time-to-contact was greater in	Shorter time-to-contact observed in plantar fasciitis compared to the other groups during midstance may indicate

	fasciitis, runners with resolved plantar fasciitis, and healthy runners	plantar fasciitis group compared to resolved plantar fasciitis group to the anterior boundary during late stance.	reduced dynamic stability during the most stable portion of running which may lead to increased injury risk.
Kettner et al., 2025a [60]	To analyze the effects of running shoes with different stack heights during treadmill running at different speeds with a 3D full-body UCM model	With increasing speed from 10 to 15 km/h, the joint angle coordination variability affecting the center of mass increased and UCM_{ratio} decreased independent of the shoe condition.	The variations in the tested running speeds had a greater impact on the running coordination than those of the tested shoes within the UCM framework.
Kettner et al., 2025b [21]	To investigate the effects of shoes with different stack heights on running style and stability during level running at different speeds.	High shoes led to a longer foot eversion during stance compared to Medium shoes. In addition, the local dynamic stability of the hip decreased with High shoes in comparison with Low shoes.	The highest stack height resulted in changes related to instabilities.
Law et al., 2018 [70]	To better understand the separate perceptual and dynamic stability effects of varying shoe top visual patterns on both walking and running activities	Participants wearing shoes with plain and horizontal striped patterns demonstrated smaller maximum eversion velocity, when compared with running with shoes having outward and vertical striped patterns.	Certain visual patterns on the tops of shoes influence the wearers' width perception and locomotion in ways that affect ankle stability during running.
Liew et al., 2024 [89]	To investigate if the step-to-step variability in leg length and angle are exploited to stabilize the center of mass trajectory during human running	Index of motor abundance in AP and vertical directions were largely destabilizing and always stabilizing, respectively.	Two simple parameters from a simple spring-mass model, leg length and angle, can explain the control behind running.
Look et al., 2013 [74]	To explore the effects of speed, stability, and leg prosthesis use in the dynamics of running	Stability decreased at faster speeds for all runners. The lower limb dynamics were less stable for the affected leg of runners with an amputation than for their unaffected leg and less stable than either leg of the non-amputee runners. The center of mass dynamics of non-amputee runners was less stable than for runners with an amputation.	Asymmetries may lead to instability in the leg dynamics that is compensated for by increased control of the center of mass.
Mehdizadeh et al., 2014 [85]	To investigate the effect of running speed on local dynamic stability of forward and backward running on a treadmill	Short term MLE increased with participant speed meaning that local dynamic stability decreased with increasing speed. Long-term largest MLE, however, remained unaffected as speed increased.	Slow running is more stable than fast running.
Mehdizadeh et al., 2016 [80]	To investigate the effect of speed as a constraint on system stability during running in forward, backward and lateral directions in skilled athletes	Speed did not change local and orbital dynamic stabilities in any of running patterns. However, both local and orbital dynamic stability were higher in the secondary plane of progression.	Less active control is required in the secondary plane of progression.

Möhler et al., 2019 [22]	To analyze the effects of fatigue on the synergy structure stabilizing the center of mass trajectory during running and to perform this analysis with two different models to better understand their influence on the outcome of the UCM analysis	Fatigue-induced changes in the UCM structure could only be detected using the 3D model. UCM_{ratio} did not change, but UCM increased during flight phase.	Only minor changes with fatigue were detected using the 3D subject-specific model. As the 2D model was not able to detect these changes, our study emphasizes that future studies on the effects of fatigue should focus on 3D analyses.
Möhler et al., 2020 [23]	To analyze the effects of expertise on the synergy structure stabilizing the center of mass trajectory in two groups: experienced runners compared to novices	No differences in UCM_{ratio} between the two groups. In novices, $UCM_{parallel}$ and UCM were higher compared to experts at 15 km/h.	Experts adopted a more consistent running style. The stride-to-stride variability was diminished but not abolished. This difference was only visible at 15 km/h.
Möhler et al., 2022 [90]	To analyze the influence of fatigue on the stride-to-stride motor variability structure that stabilizes the center of mass trajectory in novice runners	Motor variability increased with fatigue but UCM_{ratio} did not change.	Control of the center of mass decreased, whereas the stability was not affected.
Ogaya et al., 2024 [79]	To investigate the change in joint angle and its variability during running following trunk muscle training	Following the trunk training intervention, MLE flexion-extension angle increased.	The trunk training promoted adjustments to lumbar movement and altered the movement patterns of the participants' lower limbs during running.
Promsri, 2022 [77]	To investigate running stability in terms of the difference in neuromuscular control of the main movement synergies of treadmill running between male and female recreational runners	Female runners have lower stability in the mid-stance-phase movements.	Inherent impacts of sex differences in running stability should be considered for sports-related injury prevention and rehabilitation.
Rethwilm et al., 2021 [71]	To investigate how children and adolescents with cerebral palsy regulate the margin of stability during running	The ML margin of stability was higher in the cerebral palsy group and was unchanged during running.	Young people with cerebral palsy manage to maintain their medio-lateral margin of stability during running, however, with higher safety margins compared to typically developed.
Santuz et al., 2018 [18]	To improve our understanding of the mechanisms underlying the neuromuscular control of human unsteady locomotion by investigating running on an even and an uneven surface treadmill	Decreased stability when switching from even to uneven surface running.	Humans adjust their motor control strategies' timing to deal with unsteady locomotion.

Schütte et al., 2016 [68]	To investigate outdoor surface effects on dynamic stability and loading during running using triaxial trunk accelerometry	Compared to concrete road, woodchip trail led to higher AP ratio of acceleration RMS, lower ML inter-step and inter-stride regularity.	Woodchip trails disrupt aspects of dynamic stability that are detectable using a single trunk accelerometer.
Schütte, Sackey, et al., 2018 [67]	To test a cost of instability hypothesis that proposes a link between a runner's stability and running economy and that this link can be assessed using measures derived from wearable triaxial trunk accelerometry	Three accelerometry based measures could explain an additional 10.4% of interindividual variance in energetic cost after controlling for body mass, attributed to AP stride regularity, AP RMS ratio, and mediolateral sample entropy.	Wearable trunk accelerometry is a useful tool for understanding energetic cost of running and running stability is important for economy in recreational runners.
Schütte, Seerden, et al., 2018 [19]	To determine the influence of outdoor running fatigue and medial tibial stress syndrome on both dynamic loading and dynamic stability derived from trunk and tibial accelerometry	In medial tibial stress syndrome only, running fatigue was associated with decreased mediolateral sample entropy.	Entire acceleration waveform signals reflecting ML trunk control is related to medial tibial stress syndrome history, a compensation that went undetected in the non-fatigued running state.
Sterzing et al., 2015 [59]	To examine the influence of systematically varied crash-pad horizontal extent and vertical position at the posterior-lateral midsole on heel-toe running biomechanics and perception	Rearfoot motion variables indicated biomechanical effects on running shoe stability induced by crash-pad size and position.	Crash-pads applied in this research influenced rearfoot stability characteristics during running.
von Diecken et al., 2025 [20]	To investigate the relationship between dynamic stability and running economy in a sample of trained male and female runners during treadmill running at three individualized speeds	The MLE calculated from a multivariate state-space is negatively associated with cost of transport.	Runners with lower dynamic stability exhibit better running economy.
Walsh, 2021 [83]	To investigate the effects of increasing task demands on the neural dynamics of motor control during running by employing preferred and faster than preferred speeds and cognitive dual task conditions	Increasing locomotion speed decreased stability of center of mass motion with a concurrent decrease in synergy stability. Vertical center of mass motion was less stable but with no change in AP or ML directions in dual task locomotion. Muscle synergies were also more stable in dual task conditions.	Increased cognitive demands lead to more stable neuromotor commands possibly to maintain local stability of center of mass motion in the AP and ML directions.
Wang & Yuan, 2024 [65]	To analyze the differences in lower limb kinematics, dynamics, and energy absorption under walking and running conditions between patients with functional ankle instability wearing elastic ankle braces and those without elastic ankle supports	Peak of ankle varus angle, speed of ankle varus angle in ankle brace group were lower than those in control group, while peak of ankle valgus torque in ankle group was obviously more than that in control group.	The protective effects of elastic ankle brace for functional ankle instability were more prominent in contrast to that without ankle brace support.

Xiang et al., 2022 [82]	To determine foot pronation changes during and after 10 km treadmill running by comparing the pronated angle, pressure beneath the foot arch, and peak resultant acceleration	At the dorsum of the foot, MLE after 10 km running decreased 0.28 bit/s compared with MLE after 7 km running. In the tibia, MLE after 4 km and 10 km running was decreased.	Foot pronation during distance running increased the distal tibia peak impact acceleration but did not increase running instability.
Zhang et al., 2024 [72]	To investigate the potential biomechanical risk factors associated with ankle pain at different locations and to develop a risk prediction nomogram model for anterolateral, ML, and posterolateral ankle pain in runners with chronic ankle instability	ML ankle pain might be explained by greater ankle inversion angle and GRF. Moreover, posterolateral ankle pain was predicted by increased ankle adduction angle, increased GRF, and decreased dynamic stability.	To prevent ankle pain, runners with chronic ankle instability should be encouraged to focus on improving the neuroreceptor sensitivity of the gastrocnemius muscles, and retraining their energy absorption patterns.

Abbreviations: AP, anteroposterior; CNS, central nervous system; DFA, detrended fluctuation analysis; GRF, ground reaction force; ICC, intraclass correlation coefficient; IMU, inertial measurement unit; ML, mediolateral; MLE, maximum Lyapunov exponent; RMS, root mean square; SD, standard deviation; UCM, uncontrolled manifold.

Supplementary Table 4 Reporting quality assessment of the included studies. Each study was evaluated using the nine quality assessment criteria defined in Table 2. Items Q1–Q9 were scored as 0, 1, or 2, indicating that the criterion was not fulfilled, partly fulfilled or insufficiently reported, or clearly fulfilled, respectively. The total score was calculated as the sum of all item scores, with a maximum possible score of 18. Quality is expressed as a percentage of the maximum score.

Study	Q1	Q2	Q3	Q4	Q5	Q6	Q7	Q8	Q9	Total score	Quality (%)
Agresta et al., 2019 [78]	2	0	1	1	1	1	1	1	0	8	44
Aristizábal Pla et al., 2021 [66]	2	0	2	1	1	1	1	2	0	10	56
Arshi et al., 2015 [73]	2	0	2	1	1	1	1	2	0	10	56
Ataabadi et al., 2021* [61]	2	2	2	1	1	1	1	2	0	12	67
Blum et al., 2011 [75]	2	0	1	0	0	1	1	1	0	6	33
Cerrito et al., 2025 [87]	2	2	2	2	1	1	1	2	0	13	72
Dias et al., 2025 [88]	2	0	2	1	1	1	1	1	0	9	50
Ekizos et al., 2017 [14]	2	0	1	1	1	1	2	2	0	10	56
Ekizos et al., 2018 [81]	2	0	1	1	1	1	1	2	2	11	61
Fohrmann et al., 2023 [13]	2	0	1	2	1	1	2	2	2	13	72
Fohrmann et al., 2025 [76]	2	2	1	2	2	1	2	2	0	14	78
Frank et al., 2019 [17]	2	0	2	1	1	1	1	1	0	9	50
Garofolini et al., 2024 [91]	2	0	1	1	1	1	2	1	0	9	50
Hagen et al., 2025 [58]	2	2	1	1	0	1	1	1	0	9	50
Hilário et al., 2022 [69]	2	0	1	1	1	1	1	1	0	8	44
Hoenig et al., 2019 [86]	2	0	1	2	2	1	2	2	0	12	67
Hollander et al., 2021 [84]	2	0	1	2	0	1	2	2	0	10	56
Iosa et al., 2013 [62]	2	0	1	1	1	1	2	2	0	10	56
Jordan et al., 2009 [63]	2	0	1	0	2	1	1	1	0	8	44
Kelly et al., 2022 [64]	2	2	2	1	0	1	2	1	0	11	61
Kettner et al., 2025a [60]	2	0	2	1	1	1	1	2	0	10	56
Kettner et al., 2025b [21]	2	0	2	1	1	1	2	2	0	11	61
Law et al., 2018 [70]	2	0	1	1	1	1	1	1	0	8	44
Liew et al., 2024 [89]	2	0	2	1	0	1	2	1	0	9	50
Look et al., 2013 [74]	2	0	1	1	1	1	1	1	0	8	44
Mehdizadeh et al., 2014 [85]	2	0	1	1	1	1	2	2	0	10	56
Mehdizadeh et al., 2016 [80]	2	0	2	1	1	2	2	1	0	11	61

Möhler et al., 2019 [22]	2	0	2	1	1	2	2	2	0	12	67
Möhler et al., 2020 [23]	2	0	2	1	1	2	2	2	0	12	67
Möhler et al., 2022 [90]	2	0	2	1	2	2	2	2	0	13	72
Ogaya et al., 2024 [79]	1	0	1	1	1	2	1	1	0	8	44
Promsri, 2022 [77]	2	2	2	1	2	2	1	1	0	13	72
Rethwilm et al., 2021 [71]	2	0	1	1	0	1	2	1	0	8	44
Santuz et al., 2018 [18]	2	0	1	1	1	1	2	2	0	10	56
Schütte et al., 2016 [68]	2	0	2	2	1	1	1	1	0	10	56
Schütte, Sackey, et al., 2018 [67]	1	0	1	2	2	1	1	1	0	9	50
Schütte, Seerden, et al., 2018 [19]	2	2	2	1	1	2	1	1	0	12	67
Sterzing et al., 2015 [59]	2	0	2	1	1	2	2	1	0	11	61
von Diecken et al., 2025 [20]	2	0	2	1	0	2	2	2	0	11	61
Walsh, 2021 [83]	2	0	1	1	1	1	2	2	0	10	56
Wang & Yuan, 2024 [65]	1	0	1	2	1	1	2	1	0	9	50
Xiang et al., 2022 [82]	2	1	1	1	1	2	2	2	0	12	67
Zhang et al., 2024 [72]	2	2	1	1	2	1	2	1	0	12	67
Mean	1.93	0.40	1.44	1.14	1.00	1.23	1.53	1.49	0.09	10.26	57

Abbreviations: Q, quality assessment item.
